# Salicylic Acid Targets Protein Phosphatase 2A to Attenuate Growth in Plants

**DOI:** 10.1016/j.cub.2019.11.058

**Published:** 2020-02-03

**Authors:** Shutang Tan, Melinda Abas, Inge Verstraeten, Matouš Glanc, Gergely Molnár, Jakub Hajný, Pavel Lasák, Ivan Petřík, Eugenia Russinova, Jan Petrášek, Ondřej Novák, Jiří Pospíšil, Jiří Friml

**Affiliations:** 1 Institute of Science and Technology Austria (IST Austria), Am Campus 1, 3400 Klosterneuburg, Austria; 2 Department of Applied Genetics and Cell Biology, University of Natural Resources and Life Sciences (BOKU), Muthgasse 18, 1190 Vienna, Austria; 3 Department of Experimental Plant Biology, Faculty of Science, Charles University, Viničná 5, 128 44 Prague 2, Czech Republic; 4 Laboratory of Growth Regulators, The Czech Academy of Sciences, Institute of Experimental Botany & Palacký University, Faculty of Science, Šlechtitelů 27, 783 71 Olomouc, Czech Republic; 5 Department of Organic Chemistry, Faculty of Science, Palacký University, tř. 17. listopadu 1192/12, CZ-771 46 Olomouc, Czech Republic; 6 Department of Plant Biotechnology and Bioinformatics, Ghent University, 9052 Ghent, Belgium; 7 Center for Plant Systems Biology, VIB, 9052 Ghent, Belgium; 8 Institute of Experimental Botany, The Czech Academy of Sciences, Rozvojová 263, 165 02 Prague 6, Czech Republic

**Keywords:** salicylic acid, protein phosphatase 2A, PP2A, phosphorylation, NPR1, immunity, auxin, auxin transport, PIN, gravitropism

## Abstract

Plants, like other multicellular organisms, survive through a delicate balance between growth and defense against pathogens. Salicylic acid (SA) is a major defense signal in plants, and the perception mechanism as well as downstream signaling activating the immune response are known. Here, we identify a parallel SA signaling that mediates growth attenuation. SA directly binds to A subunits of protein phosphatase 2A (PP2A), inhibiting activity of this complex. Among PP2A targets, the PIN2 auxin transporter is hyperphosphorylated in response to SA, leading to changed activity of this important growth regulator. Accordingly, auxin transport and auxin-mediated root development, including growth, gravitropic response, and lateral root organogenesis, are inhibited. This study reveals how SA, besides activating immunity, concomitantly attenuates growth through crosstalk with the auxin distribution network. Further analysis of this dual role of SA and characterization of additional SA-regulated PP2A targets will provide further insights into mechanisms maintaining a balance between growth and defense.

## Highlights

• SA modulates root development independently of NPR1-mediated canonical signaling
• SA attenuates growth through crosstalk with the auxin transport network
• SA upregulates the phosphorylation status of PIN auxin efflux carriers through PP2A
• SA directly targets A subunits of PP2A, inhibiting the activity of the complex

## Graphical Abstract

**Figure:**
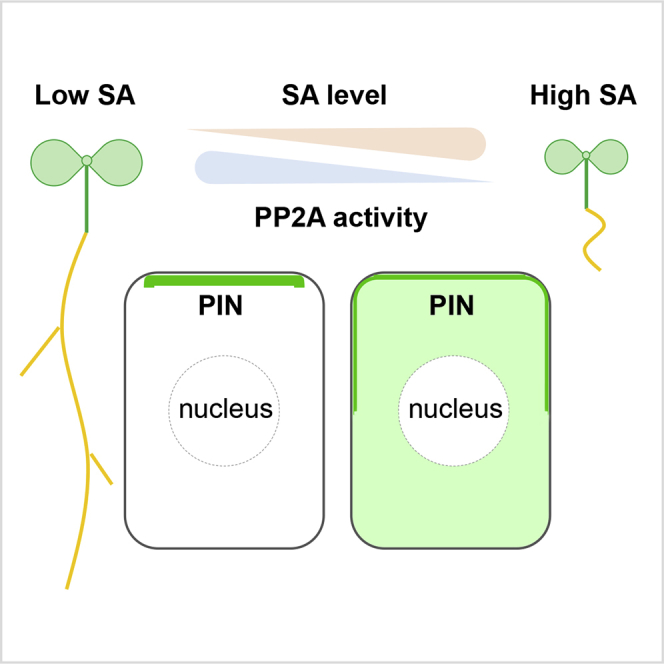

## Teaser

Besides the canonical role in immunity, the defense hormone salicylic acid also shapes plant growth and development. Tan et al. show that salicylic acid regulates *Arabidopsis* growth through directly binding to A subunits of the PP2A enzyme and thereby modulating its activity.

## Introduction

Life of multicellular organisms is a permanent trade-off to allocate resources between growth and defense against pathogens. Salicylic acid (SA) is a classical plant hormone traditionally connected with plant immunity, and its levels increase in response to pathogen attack [1]. SA functions as an endogenous signal mediating local and systemic defense responses against pathogens by upregulating the production of pathogenesis-related (PR) proteins. The best characterized components of the SA immunity pathway are the NPR (NONEXPRESSOR OF PR GENES) proteins that include four close isoforms, NPR1–NPR4 [2, 3, 4]. Following increase in SA levels, NPR1 translocates from cytoplasm into nucleus [5, 6, 7], thereby allowing binding to the downstream transcription factors and regulation of the expression of downstream genes [8]. NPR1, together with NPR3/NPR4, were shown to be bona fide SA receptors for the immune pathway [7, 9, 10]. NPR1 functions as a transcriptional activator, whereas NPR3 and NPR4 are transcriptional repressors, all working independently and harmoniously to regulate the expression of downstream genes [7].

Much less understood is the role of SA beyond plant immunity, in particular in modulating plant growth and development. SA has been implicated in the regulation of photosynthesis, respiration, flowering, senescence, seed germination, and growth. Nevertheless, whether SA signaling for these functions depends on the NPR-mediated pathway or other, so far molecularly uncharacterized mechanism(s) remains unclear [8, 11, 12, 13, 14, 15]. Biochemical approaches have identified numerous potential SA binding proteins (SABPs), but their potential roles in SA physiological functions remain unclear [16, 17, 18, 19].

SA, similarly to other endogenous signals in plants, executes its effect in concert with other plant hormones. In particular, the SA-auxin signaling crosstalk has been proposed to be important for SA roles in balancing plant defense and development [15]. This notion was strengthened by the observation that SA affects the constitutive subcellular dynamics of PIN (PIN FORMED) auxin transporters [14, 20], which are important regulators of many developmental processes [21]. Nonetheless, the physiological relevance of this SA regulation or the underlying signaling mechanism remains elusive.

Here, we demonstrate an alternative SA signaling mechanism, by which SA, in addition to activating plant immunity, attenuates root growth through regulating PIN-dependent auxin distribution network.

## Results

### SA Regulates Root Growth Independently of Canonical NPR Receptors

**Figure 1:**
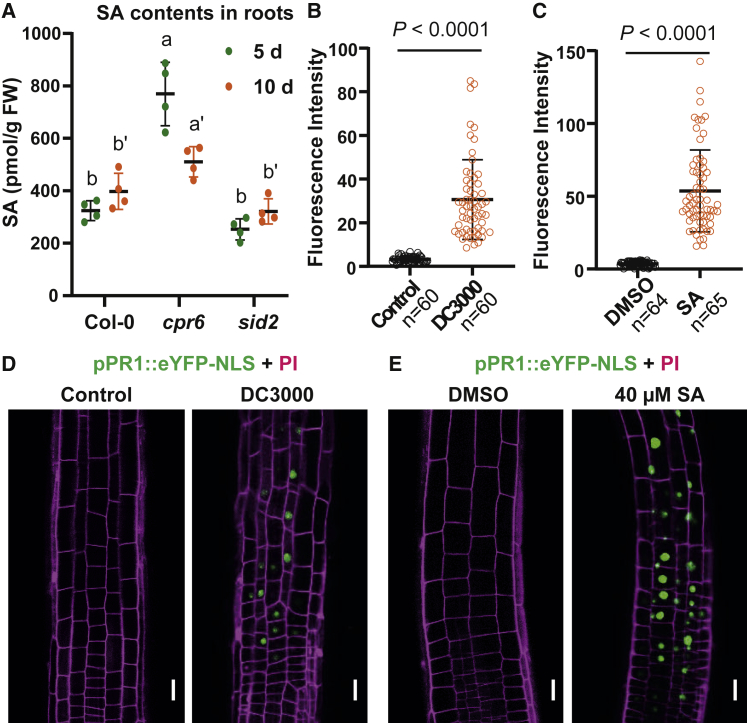
Pathogen-Induced SA Response in Roots, Revealed by the *pPR1::eYFP-NLS* Reporter (A) SA contents in the roots of 5- or 10-day-old seedlings of Col-0, *cpr6*, and *sid2* (*sid2-3*) measured by LC/MS-MS. n = 4 replicates, with multiple seedlings for each. Dots represent individual values, and lines indicate mean ± SD. Different letters represent significant difference; p < 0.05; by one-way ANOVA with a Tukey multiple comparison test. (B–E) Induced *pPR1::eYFP-NLS* expression by *P. syringae* DC3000 (B and D) or SA (C and E) in roots. (B and D) 5-day-old *pPR1::eYFP-NLS* seedlings were treated with *P. syringae* DC3000 (optical density 600 [OD_600_] = 0.01, ∼5 × 10^6^ colony-forming units [CFUs]/mL) or with resuspension buffer (control) for 48 h and were then imaged by confocal laser scanning microscope (CLSM). (C and E) For SA treatment, 5-day-old *pPR1::eYFP-NLS* seedlings were transferred to plates with DMSO or 40 μM SA for 24 h and were then imaged by CLSM. Scale bars, 10 μm. For quantification, the average GFP florescence of 5–10 representative cells from 10 seedlings for each treatment was measured by Fiji. The data points were shown as dot plots. Dots represent individual values, and lines indicate mean ± SD. p values were calculated by a two-tailed t test. See also Figure S1.

The majority of SA physiology studies have focused on adult-stage shoots and so far it remains unclear whether there are significant levels of SA in the root. Therefore, we examined the SA contents by liquid chromatography-tandem mass spectrometry (LC-MS/MS) first. SA production is typically highly elevated after pathogen attack [22], and thus, the basal SA levels in the roots were relatively low but detectable (Figure 1A). There was a small decrease in the SA-biosynthesis-deficient mutant, *sid2-3* [1], and a corresponding increase in the SA overproduction mutant, *cpr6* [23]. Moreover, using *pPR1::eYFP-NLS* reporter line for the NPR1 pathway [24], we detected an induced *PR1* expression in both shoots (Figures S1A–S1D) and roots (Figures 1B–1E) following treatment with either a plant pathogen, *Pseudomonas syringe* DC3000 (Figures 1B and 1D), or SA (Figures 1C and 1E), confirming that pathogen- or SA-mediated activation of NPR1 pathway occurs also in roots.

**Figure 2:**
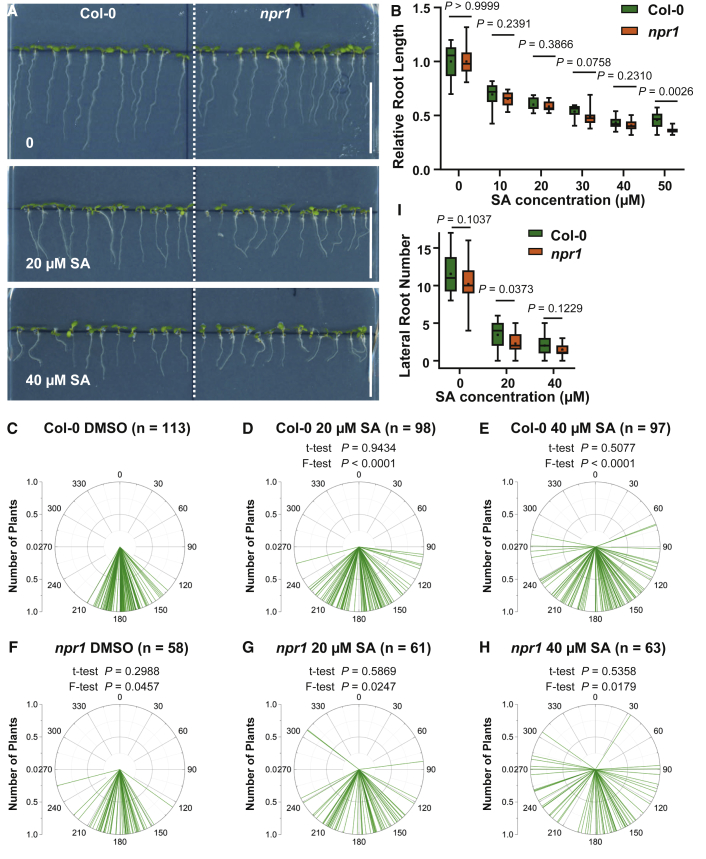
SA Regulates Root Growth and Development in a *NPR1*-Independent Manner (A) Representative images showing the morphological changes of Col-0 and *npr1* under SA treatment. DMSO is the solvent control. Scale bars, 2 cm. (B) SA inhibited the primary root elongation in a *NPR1*-independent manner. Root length of 7-day-old Col-0 and *npr1* seedlings grown on MS plates with different concentrations of SA was measured. Relative length was calculated by dividing the values with the root length at SA = 0. Boxplots show the first and third quartiles, with whiskers indicating maximum and minimum, the line for median, and the black dot for mean. n = 11–28; p values were calculated by a two-tailed t test for indicated pairs of Col-0 and *npr1* at a certain concentration of SA. (C–H) SA interfered with root gravitropism independently of *NPR1*. Root tip angles of 7-day-old Col-0 (C–E) and *npr1* (F–H) seedlings were measured and shown as polar bar charts. Two-tailed t tests were performed to indicate the difference of mean value, and F-tests indicate the difference of variances. For Col-0, SA treatments were compared with the DMSO control, and the *npr1* groups were compared with Col-0 under the same SA treatment, respectively. (I) Inhibition of lateral root formation by SA does not involve *NPR1*. The number of emerged lateral roots for 10-day-old plants was counted. n = 20–25. p values were calculated by a two-tailed t test. See also Figure S1.

Given detectable levels of SA in roots and previous indications about a physiological role of SA in roots [14, 25], we examined the effect of exogenously applied SA on root growth. Compared to the control conditions, seedlings growing on 20 or 40 μM SA exhibited shorter (Figures 2A and 2B) and partially agravitropic roots (Figures 2C–2H), as well as fewer lateral roots (Figure 2I). Two inactive SA isomers, 3-hydroxybenzoic acid (3-OH-BA) and 4-hydroxybenzoic acid (4-OH-BA) [26], did not show any obvious effects at comparable concentrations (Figures S1E–S1J). These observations show that SA impacts root development at concentrations equal to or below those established in shoots [7] and its activity is specific to its active structure.

Next, we addressed the requirement of the SA receptors, NPR1/NPR3/NPR4, which are well established in the immune response, for the observed root response [2, 3, 4, 7, 10]. NPR1 is a central regulator of the canonical immune pathway, and the downstream transcriptional responses are completely blocked by *npr1* deficiency [3]. Unexpectedly, the well-characterized corresponding mutants *npr1*, *npr3,4* double, and *npr1,3,4* triple mutants did not show a decreased sensitivity to SA in terms of root elongation, gravitropic growth, and lateral root formation (Figures 2B–2I and S1K–S1R). It is noteworthy that the *npr1,3,4* triple mutant exhibited even a pronounced SA-hypersensitive phenotype (Figures S1K–S1R), which might come from downregulation of multiple genes involved in auxin biosynthesis, transport, or signaling.

In conclusion, SA regulates multiple aspects of root development by a signaling mechanism not requiring the established NPR receptors.

### SA Regulates PIN-Dependent Auxin Transport and PIN2 Phosphorylation

**Figure 3:**
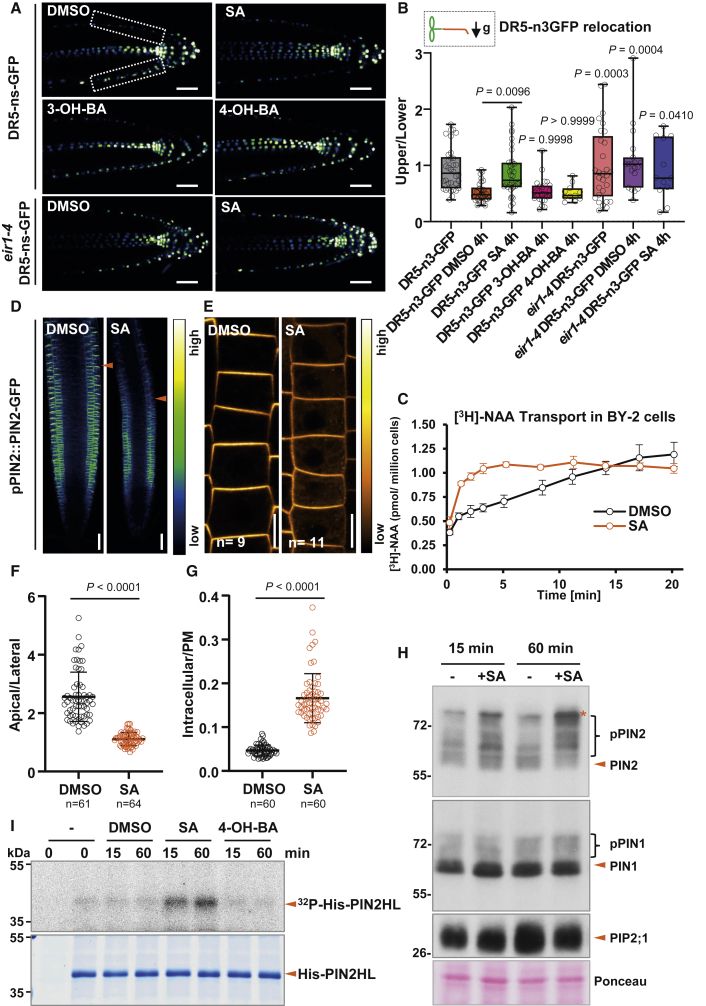
SA Regulates Auxin Transport via Modulating PIN2 Phosphorylation (A) SA inhibited the relocation of DR5-n3GFP. 5-day-old *DR5v2* and *eir1-4 DR5v2* seedlings were transferred to different plates with DMSO, 40 μM SA, 40 μM 3-OH-BA, or 40 μM 4-OH-BA, respectively, and then turned 90 degrees for gravistimulation. After 4 h, the roots were imaged by CLSM. The GFP channel (DR5-n3GFP) was shown. Scale bars, 10 μm. (B) The ratio of fluorescence between the upper side and the lower side was measured, as shown in (A). n = 34, 30, 35, 24, 11, 29, 19, and 12, respectively. p values are calculated by a two-tailed t test, comparing different datasets with the DR5-n3-GFP DMSO control (t = 4 h), as shown with the horizontal line in the case of DR5-n3-GFP SA 4 h. (C) SA treatment increased the accumulation of [^3^H]-NAA in tobacco BY-2 cells, suggesting a decrease in auxin export. DMSO and 200 μM SA were added to the cell culture and then the radioactivity inside of cells was measured at indicated time points after the addition of [^3^H]-NAA to the DMSO- and SA-treated cell cultures. n = 3. (D–G) SA treatment impaired the polar localization and promoted the internalization of PIN2-GFP in root epidermis (D and E). *pPIN2::PIN2-GFP* seedlings were grown on plates with DMSO and 40 μM SA for 4 days and were then imaged by CLSM. Scale bars, 20 μm (D) and 10 μm (E), respectively. Arrowheads in (D) indicate the beginning of root transition zone. (F) The intensity ratio of apical/lateral was measured by Fiji to assess PIN2 polarity. (G) Quantification of the PIN2-GFP intensity ratio of intracellular/PM. (F and G) Dots represent individual values, and lines indicate mean ± SD. p values are calculated by a two-tailed t test. (H) SA treatment enhanced the phosphorylation of PIN2. Roots of 7-day seedlings were treated with DMSO or 40 μM SA for 15 min and 60 min and then analyzed by western blot with an anti-PIN2 antibody (upper panel). Phosphorylation of the multiple phosphorylation sites in PIN2 causes slower migrating species. The more highly phosphorylated, the slower the migration. The same membrane was stripped and detected by anti-PIN1 (second panel) and anti-PIP2;1 (third panel) antibodies, sequentially. The molecular weight (MW) of PIN2 and PIN1 is 69 and 67 kDa, respectively. For unknown reasons, PIN2 runs faster than expected, perhaps due to incomplete denaturing when heated only at 50°C. The shifted bands indicate the phosphorylated PIN proteins. Bottom panel: Ponceau staining is shown. Asterisk indicates partial contribution by a non-specific band (see also in Figure 4A). (I) SA treatment increased the phosphorylation of His-PIN2-HL in plant extracts. Roots of 7-day seedlings were treated with DMSO or 40 μM SA for 15 min and 60 min, respectively, and then were subject to protein extraction. Crude plant extracts were incubated with recombinant His-PIN2-HL for 60 min with ^32^P-ATP and MgCl_2_. The first lane was without His-PIN2-HL as negative control. Reaction samples were analyzed by SDS-PAGE and the subsequent autoradiography. Upper panel: autoradiography is shown; lower panel: Coomassie Brilliant Blue (CBB) staining is shown. See also Figures S2 and S3.

The root phenotypes generated by SA treatment are reminiscent of defective auxin homeostasis because auxin and its distribution have been shown to regulate primary root growth, gravitropic bending, and lateral root formation [21, 27]. To test the potential effect of SA on auxin response and distribution, we used an auxin-responsive marker DR5-n3GFP (the GFP channel of DR5v2) [28], which monitors auxin response in plant tissues, including the gravity-induced auxin translocation to the lower root side [28]. After 4-h gravistimulation by 90° reorientation, the seedlings treated with SA, unlike the DMSO-treated controls, failed to show a pronounced DR5-n3GFP asymmetry with the stronger signal at the lower root side, in line with the SA-induced gravitropism defect (Figures 3A, 3B, and S2A), as observed before [14]. This suggests that SA interferes with auxin distribution either at the level of transport [21] or local auxin biosynthesis [29]. Recently, SA has been proposed to increase auxin levels in root tips [30]. Nonetheless, this upregulation of iIndole-3-acetic acid (IAA) biosynthesis cannot explain the auxin-related phenotypes described here, such as agravitropic root growth and the reduced lateral root number, because increased auxin levels have rather opposite effects. It is likely that increased IAA biosynthesis after SA treatment is rather the consequence, but not the cause, of the auxin transport regulations by SA, presumably due to a feedback regulatory mechanism.

To test a possible effect of SA on auxin transport, we measured the basipetal (rootward) auxin transport in etiolated hypocotyls, which revealed that SA can inhibit the rootward transport of [^3^H]-IAA, similar to widely used PIN-dependent auxin transport inhibitors NPA (1-N-naphthylphthalamic acid) and TIBA (2,3,5-triiodobenzoic acid) (Figure S2B). With tobacco BY-2 cultured cells [31], we tested the effect of SA on transport of different auxin analogs, [^3^H]-NAA and [^3^H]-2,4-D. SA treatment increased the cellular accumulation of [^3^H]-NAA (Figure 3C), but not of [^3^H]-2,4-D or [^3^H]-BA (Figures S2C and S2D). Despite possible effect on auxin metabolism, this selective effect of SA on accumulation of NAA, which is a good substrate of PIN auxin exporters [32], strongly suggests a regulatory role of SA in PIN-dependent auxin transport. Overall, these observations show that SA, exhibiting distinct activities for different tissues, directly or indirectly regulates auxin transport.

To investigate the mechanism underlying the role of SA in regulating root growth and development, we focus on the root gravitropic phenotype. PIN2 and AUX1 auxin transporters play a prominent role in shootward auxin transport in the root and thus in the auxin redistribution during the gravitropic response [33, 34, 35, 36, 37]. Therefore, we analyzed the response of *eir1-4* [36] loss-of-function mutant, which exhibits strongly agravitropic roots. After SA treatment, *eir1-4* showed a slight SA hypersensitivity in primary root elongation but no further enhancement of the gravitropic defect at 40 μM SA (Figures S2E–S2J). These observations suggest that SA acts in the gravitropic response through the auxin efflux carrier PIN2. Using the *eir1-4 DR5-n3-GFP* cross, we could not see gravity-induced DR5-n3-GFP asymmetry and SA treatment had no additional effect (Figures 3A and 3B). Furthermore, we examined the SA effect on the localization of these proteins using *pAUX1::AUX1-YFP* and *pPIN2::PIN2-GFP* marker lines. Whereas we observed no obvious effect of SA treatment on AUX1-YFP except a slight decrease in the overall intensity (Figures S2K–S2M), PIN2-GFP incidence in the plasma membrane of the root epidermis cells upon SA treatment was visibly decreased with higher intracellular signal and less pronounced polar distribution as compared to the control (Figures 3D–3G).

Reversible phosphorylation plays an important role in regulating PIN polarity, subcellular dynamics, and activity. PIN proteins can be phosphorylated by multiple kinases, most prominently PID (PINOID)/WAGs (WAVY ROOT GROWTHs), D6PK/D6PKLs, and PAX (PROTEIN KINASE ASSOCIATED WITH BRX), and dephosphorylated by various phosphatases, including protein phosphatase 2A (PP2A), PP1, and PP6 [38, 39, 40]. We examined the PIN2 phosphorylation status by western blot. When roots were extracted with a protocol [36, 41] specifically modified to preserve phosphorylation, PIN2 appeared as a smear of bands (Figure 3H). Phosphatase treatment shifted the slower migrating bands toward the faster migrating band at the base of the smear (Figure S3A), indicating that the upper parts of the smear comprise phosphorylated species. We found that SA treatment led to a more pronounced shift of PIN2 protein mobility to slower migrating species than seen in control, indicating an increase in phosphorylation status. This occurred as rapidly as after 15 min and more pronounced after 60 min (Figures 3H and S3A). To confirm the SA effect on the change of PIN2 phosphorylation, we expressed and purified the PIN2 hydrophilic loop with His tag (His-PIN2HL) and incubated it with the protein extract from seedlings treated with SA or the inactive isomers in a ^32^P-ATP phosphorylation reaction (Figure 3I). There was more phosphorylation of the His-PIN2HL with SA. This confirmed that SA treatment led to an increase in PIN2 phosphorylation level, either through stimulating phosphorylation or suppressing dephosphorylation.

Taken together, the physiological, microscopic, and biochemical observations collectively suggest that SA regulates PIN-dependent auxin transport, presumably by changing the phosphorylation status and thus the cellular localization and the activity of PIN proteins. Such mechanism would explain the observed physiological SA effects on root development.

### PP2A Is Required for SA Regulation of PIN2 Phosphorylation and Root Development

To gain insight into the mechanism by which SA modulates PIN phosphorylation and root development, we focused on the potential regulators of PIN phosphorylation. Of those, the A subunit of PP2A (PP2AA1/RCN1, ROOTS CURL IN NPA1), an established regulator of PIN phosphorylation and auxin transport [42, 43], came to our attention, as it also appeared in a high-throughput proteomics study as potentially associated with SA binding [44].

**Figure 4:**
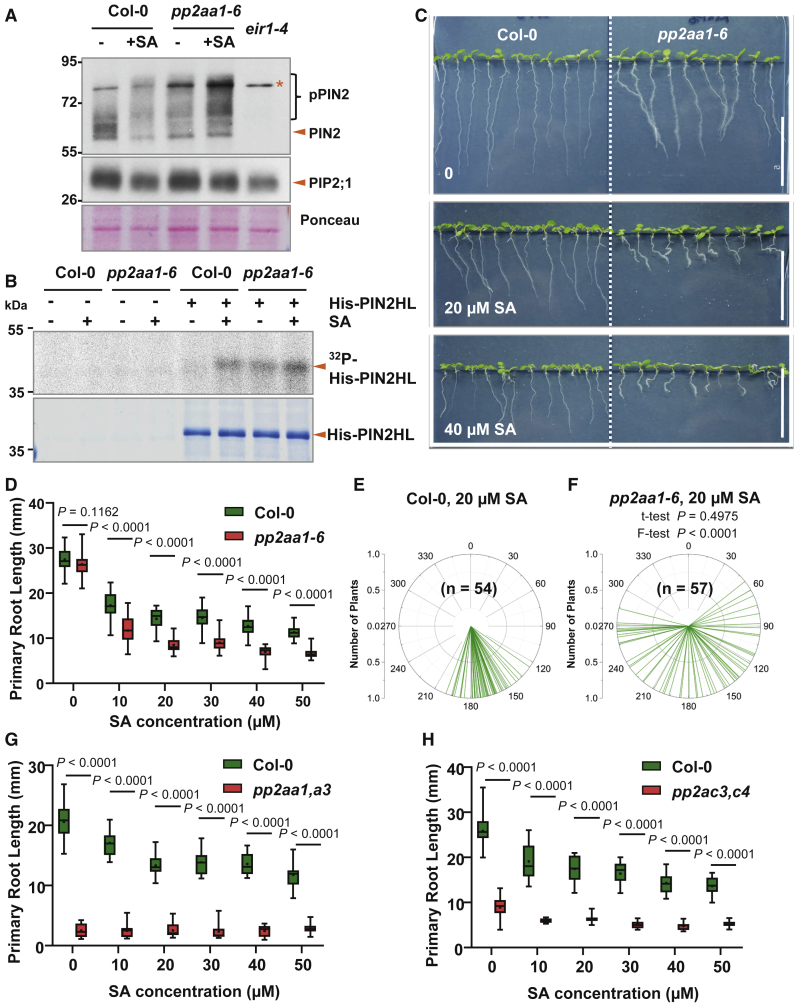
SA Functions through PP2A in Regulating Root Development (A) SA treatment promoted the phosphorylation of PIN2 in Col-0 to a similar degree as that in *pp2aa1-6*. Roots of 7-day-old Col-0 and *pp2aa1-6* seedlings were treated with DMSO or 40 μM SA for 60 min and were then sampled for protein isolation and western blot. The shifted bands indicate the phosphorylated PIN proteins (upper panel). Asterisk indicates a non-specific band that contributes partially to the signal. The same membrane was stripped and probed with a PIP2;1 antibody to indicate the loading (upper panel). Ponceau staining is shown in the bottom panel. (B) Phosphorylation with ^32^P-ATP revealed that SA treatment increased the phosphorylation of His-PIN2-HL in Col-0, whereas this increase was attenuated in *pp2aa1-6*. Upper panel: autoradiography is shown; lower panel: CBB is shown. (C) Representative images revealing the hypersensitivity of *pp2aa1-6* to SA. Col-0 and *pp2aa1-6* seedlings were grown on plates with SA. Scale bars, 2 cm. (D) *pp2aa1-6* was hypersensitive to SA in root growth inhibition. Col-0 and *pp2aa1-6* seedlings grew on plates with SA for 7 days and then the primary root length was measured. n = 11–28. p values were calculated by a two-tailed t test for indicated pairs of Col-0 and *pp2aa1-6* at a certain concentration of SA. (E and F) *pp2aa1-6* was hypersensitive to SA in terms of interfering with root gravitropism. Col-0 (E) and *pp2aa1-6* (F) seedlings grew on plates containing different concentrations of SA for 7 days, and the root tip angles were measured by ImageJ and shown as polar bar charts. p values were calculated by a two-tailed t test in (E) and (F) and indicate differences of variances by a further F-test in (F). (G) The *pp2aa1, a3* double mutant exhibited decreased sensitivity to SA. Col-0 and *pp2aa1, a3* seedlings grew on plates with SA for 7 days and then the primary root length was measured. n = 11–25. p values were calculated by a two-tailed t test for indicated pairs of Col-0 and *pp2aa1, a3* at the given concentration of SA. (H) The *pp2ac3, c4* double mutant exhibited decreased sensitivity to SA. Col-0 and *pp2ac3, c4* seedlings grew on plates with SA for 7 days and then the primary root length was measured. n = 10–21. p values were calculated by a two-tailed t test for indicated pairs of Col-0 and *pp2ac3, c4* at the given concentration of SA. See also Figures S3 and S4.

We first tested whether PP2AA1 is involved in SA-induced increase of PIN phosphorylation status. Western blot showed that SA treatment could increase the phosphorylation level of PIN2 in wild-type (WT), whereas in PP2AA1 loss-of-function mutant, *pp2aa1* (also known as *pp2aa1-6 and rcn1-6*), there was already a higher level of PIN2 phosphorylation, consistent with PP2AA1 involvement in PIN2 dephosphorylation (Figures 4A and S3B). This phosphorylation was still increased further by SA treatment (Figure 4A), suggesting that the other PP2AA homologs can play a role in the absence of PP2AA1. Accordingly, the ^32^P-ATP phosphorylation reaction using purified His-PIN2HL incubated with the protein extracts from SA-treated WT and *pp2aa1* seedlings (Figure 4B) confirmed at least partial PP2AA1 requirement for the SA effect on PIN2 phosphorylation.

In line with this, *pp2aa1* mutants (*pp2aa1-6* and *pp2aa1-1*) roots showed hypersensitivity to SA in terms of primary root growth and gravitropic bending (Figures 4C–4F and S3C–S3I). In addition, SA treatment at higher concentrations (50 μM) often led to a slight swelling in WT root tips, whereas in *pp2aa1*, a much stronger root tip swelling was observed even at a lower SA concentration (20 μM; Figure S3C).

**Figure 5:**
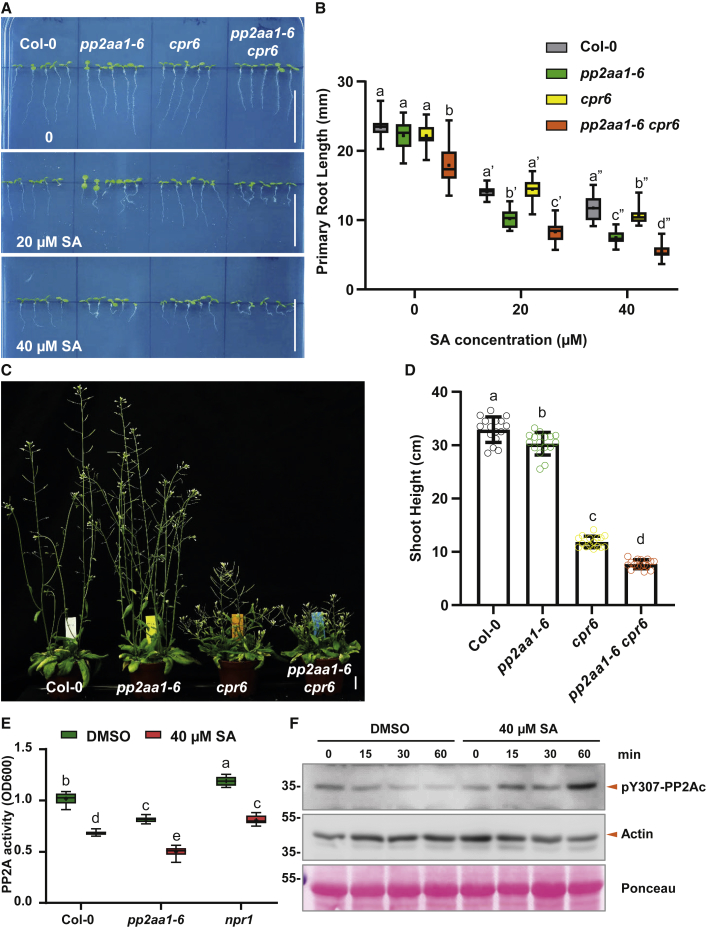
Genetic Analysis of *pp2aa1-6* and *cpr6* Mutations, and SA Inhibits PP2A Activity *In Planta* (A) Representative images showing the enhanced sensitivity of *pp2aa1-6* to SA. Col-0, *pp2aa1-6*, *cpr6*, and *pp2aa1-6 cpr6* seedlings were grown on plates with different concentrations of SA for 7 days. Scale bars, 2 cm. (B) The root growth analysis revealed that the *cpr6* mutation decreased the primary root length and increased the SA sensitivity of *pp2aa1-6*. n = 16. Different letters represent significant difference; p < 0.05; by one-way ANOVA with a Tukey multiple comparison test. (C and D) The *pp2aa1-6* mutation enhances the stunted shoot phenotype of *cpr6*. Col-0, *pp2aa1-6*, *cpr6*, and *pp2aa1-6 cpr6* plants were grown for 38 days, and representative plants are shown (C). Scale bar, 2 cm. (D) The height of plants was measured and shown as dot plots. Dots represent individual values, and lines indicate mean ± SD. n = 16. Different letters represent significant difference; p < 0.05; by one-way ANOVA with a Tukey multiple comparison test. (E) SA treatment decreased the total PP2A activity *in planta*. Col-0, *pp2aa1-6*, and *npr1* seedlings were grown on plates containing DMSO or 40 μM SA for 5 days and then sampled for protein isolation and PP2A activity measurement. n = 6. Different letters represent significant difference; p < 0.05; by one-way ANOVA with a Tukey multiple comparison test. (F) SA treatment increased the phosphorylation of the PP2A catalytic subunits (PP2Ac), suggesting the decrease in PP2A activity. 7-day-old Col-0 seedlings were treated with DMSO or 40 μM SA for 0, 15 min, 30 min, and 60 min respectively, and were then collected for protein extraction and the subsequent western blot. A pY307-PP2Ac antibody was used, 1:1,000 (upper panel). The anti-actin blot (medium panel; 1:2,000) and Ponceau staining (bottom panel) indicate the loading amounts. See also Figure S4.

PP2A is a heterotrimeric complex composed of A, B, and C subunits with three homologs for the PP2A A subunits, PP2AA1/RCN1, PP2AA2, and PP2AA3 [45]. Notably, overexpression of *PP2AA1* (*35S::myc-PP2AA1*) alone did not lead to obvious changes in SA sensitivity (Figures S3J–S3L), suggesting importance of the whole heterotrimeric PP2A holoenzyme integrity. Single mutants of *pp2aa2* and *pp2aa3* did not show any visible difference in SA sensitivity compared to WT (Figures S4A–S4C). The double mutant of *pp2aa1 pp2aa2-3* (*pp2aa1,a2*) showed a much stronger response to SA than WT or *pp2aa1/rcn1* single mutant (Figures 4D and S4D). The *pp2aa1,a3* double mutant had severe defects in growth and development with a short primary root already without any treatment (Figures S4E–S4H) [43, 45], which is reminiscent to WT treated by higher concentration of SA, and subsequent SA treatment could not further enhance this phenotype (Figure 4G). Similar results were observed for the *pp2ac3,c4* double mutant of the catalytic C subunits [46]. The roots of *pp2ac3,c4* were short without any treatment, and higher exogenous SA treatment did again not further enhance this phenotype (Figure 4H). The mutant in the regulatory subunit, *fass/tonneau2* (*ton2*), has been reported to show a similar phenotype as *pp2aa1,a3* [46]. However, *fass* [46], the double knockout mutant *pp2aa1-1 pp2aa2-1* [43, 45], and triple *pp2aa1-1 pp2aa2-1 pp2aa3-1* [43, 45] were too sick to perform meaningful SA sensitivity assays. It has been well described that these mutants exhibited severe growth defects, with swelling root morphology [45, 46], which are similar to seedlings treated with SA. Thus, loss-of-function mutants in all PP2A subunits perturbed plant sensitivity to SA in terms of root growth. Importantly, phenotypes of the stronger higher order mutants could be phenocopied by SA treatment. The SA-overproducing *cpr6* mutants show a severe dwarf phenotype [8] and increased SA levels in roots (Figure 1A) but no obvious changes in root development (Figure 5A). On the other hand, the *pp2aa1-6 cpr6* double mutant had shorter roots and increased sensitivity to SA (Figures 5A and 5B) as well as exhibited a much more severe dwarf phenotype than *cpr6* alone (Figures 5C and 5D). This provides a genetic confirmation that PP2A is involved in the SA-mediated developmental regulation.

In summary, these biochemical and genetic analyses suggest that the PP2A complex is involved in SA regulation of PIN (de)phosphorylation and root growth.

### SA Inhibits PP2A Activity

To further confirm whether SA is an endogenous regulator of PP2A, we tested the sensitivity of *pp2aa1-1* to a known PP2A inhibitor, cantharidin, that binds the C subunits in both animals and plants [45, 47, 48, 49]. When grown on media with cantharidin, WT seedlings exhibited shorter, agravitropic roots and root tip swelling as observed for SA treatment, and notably, *pp2aa1* mutants were hypersensitive to cantharidin (Figures S4I and S4J) as they were to SA. The identical physiological effects of SA to an established PP2A inhibitor and similarities between the SA effects and stronger loss-of-function phenotypes of the PP2A complex indicated that SA may act as an endogenous inhibitor of PP2A.

Therefore, we analyzed PP2A activity in the protein extracts of *Arabidopsis* seedlings using the established colorimetric method with phospho-Ser/Thr peptides as PP2A substrates [50]. This revealed that the *pp2aa1* mutant had lower PP2A activity than WT [48] and that SA treatment decreased PP2A activity in WT (Figure 5E). Notably, the *npr1* mutant defective in an established SA receptor still showed high sensitivity to SA in the PP2A activity assay (Figure 5E). Next, we established an independent method to assess the PP2A activity. In mammalian cells, phosphorylation at Tyr307 (pY307) of the catalytic subunit PP2Ac is used as a measure of PP2A activity and can be detected by a phospho-Tyr307 (pY307)-PP2Ac antibody [51]. Alignment of the five *Arabidopsis* PP2AC subunits with the human and mice homologs indicated that the antigen motif recognized by this antibody is highly conserved across different homologs (Figure S4K), which makes it feasible to use the same antibody to evaluate the PP2A activity *in planta*. The phosphorylation status of PP2ACs, monitored by this method, was robust and stable under control treatments, whereas treating seedlings with SA led to an increased PP2AC phosphorylation (Figure 5F) indicative of decreased PP2A activity.

Taken together, our physiological and biochemical observations show that SA inhibits PP2A activity, indicating that the PP2A complex could be a direct target of SA.

### SA Binds to the A Subunits of PP2A

Next, we addressed a mechanism by which SA inhibits PP2A activity. The finding that established SA receptors from the NPR family are not required for this SA effect on root growth and on PP2A activity supported a possibility that SA targets PP2A directly.

**Figure 6:**
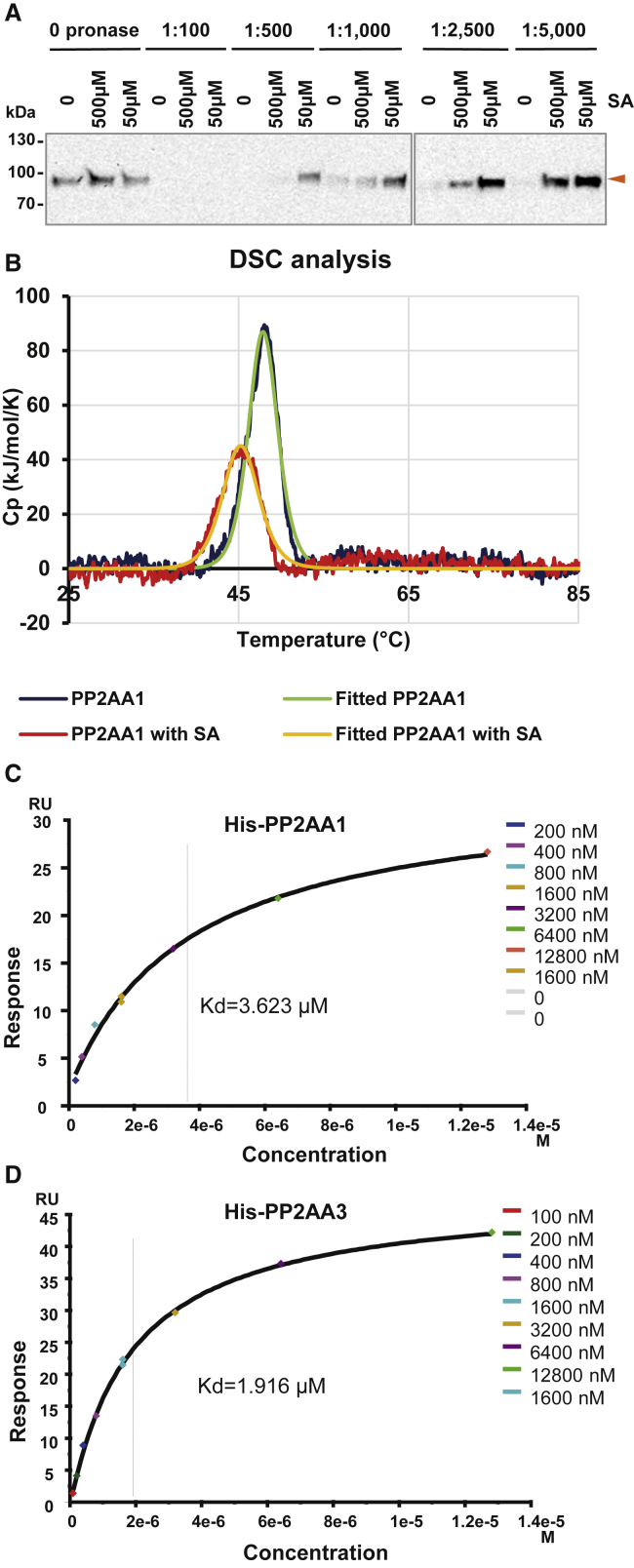
SA Binds to the A Subunit of PP2A (A) DARTS assay suggests that PP2AA1 is potential target of SA. *pPP2AA1::PP2AA1-GFP* seedlings were used for the protein isolation. Samples were treated with DMSO (mock) and SA and digested by different concentrations of Pronase. Samples were further analyzed by western blot with an anti-GFP antibody. (B) DSC analysis suggesting the potential binding of SA to recombinant His-PP2AA1. 5 μM of purified His-PP2AA1 protein was analyzed by DSC with or without 50 μM SA. Tm = 48.01°C and 45.03°C for His-PP2AA1+DMSO and His-PP2AA1+SA, respectively. (C) SPR analysis of the His-PP2AA1 and SA interaction. An active synthetic SA analog (SA-f) was immobilized on a CM-5 sensor chip, and different concentrations of His-PP2AA1 were applied. The binding curve was plotted by values at the steady state, for which the sensorgram is shown is Figure S7D. A K_D_ value of 3.623 μM was detected. (D) SPR assay reveals the binding of His-PP2AA3 to SA. The same sensor chip as above was used, and different concentrations of His-PP2AA3 were applied. The binding curve was plotted by values at the steady state, with the data points shown in the sensorgram in Figure S7E. A K_D_ value of 1.916 μM was detected. See also Figures S5, S6, and S7.

To test for a direct SA binding to PP2A, we first used the drug affinity responsive target stability (DARTS) method based on the fact that ligand binding to its protein target typically causes a conformational change, which affects the exposure of protease recognition sites and thus influences protein stability in the presence of the ligand [52]. DARTS using extracts of *pPP2AA1::PP2AA1-GFP* seedlings revealed that SA treatment led to an obvious protection of PP2AA1-GFP against Pronase (mixture of proteases) degradation, but 4-OH-BA did not (Figures 6A, S5A, and S5B). This suggests that SA targets PP2AA1 *in planta*. Notably, although SA concentration as high as 500 μM still showed pronounced protective effects toward PP2AA1-GFP, the 50 μM SA was more effective (Figure 6A). This suggests a more complicated regulatory effect of SA on PP2AA1-GFP stability for the high concentrations.

Differential scanning calorimetry (DSC) is a method to detect thermostability of a protein by measuring the heat release during denaturing [53]. We expressed and purified from *E. coli* His-PP2AA1 (Figures S5C–S5F) and used the recombinant protein for DSC. We detected a denaturing temperature (Tm) of His-PP2AA1 at 48.01°C, but following SA treatment, the Tm shifted to 45.03°C (Figure 6B), suggesting that SA treatment changed PP2AA1 stability, which might be due to conformational changes. A further control with the inactive SA isomer, 4-OH-BA, did not show any effect on PP2AA1 thermostability, confirming this specific activity of SA (Figure S5G). Usually ligand binding stabilizes the target protein [54], but in some well characterized cases, such as receptors for the plant hormone strigolactone, ligand binding caused the destabilization of the protein, which is similar to what we observed for SA and PP2AA1 [55]. Thus, DSC also supports the hypothesis of direct SA binding to PP2AA1.

To further confirm SA binding to PP2AA1 and to measure the binding affinity, we employed the surface plasmon resonance (SPR) method [56]. We first designed a SA analog with a linker, SA-f, which can be immobilized on the SPR sensory chip. A set of SA derivatives have been synthesized with modifications at the meta- and para- positions in the benzoic ring and then we first tested their bioactivity in terms of PIN2-GFP endocytic trafficking as an output of NPR-receptors-independent SA activity [14], as well as the physiological effects on root morphology that we describe here. These tests indicated that modifications at the meta- position did not affect this SA bioactivity (Figure S5H), thus identifying C-10 moiety as being most promising for further modification (Figures S6A, S6B, and S6D–S6F). For the second round, we added a -(CH_2_-)_6_-O- linker at the meta-position, SA-1∼3 (Figure S5H), with different groups at the end of the linker to mimic the matrix of sensor chips. SA-2 and SA-3 still kept the activity on PIN2-GFP trafficking (Figures S6C and S7A–S7C) and root development similar to non-modified SA (Figures S6C, S6D, and S7A–S7C). Finally, we synthesized SA-f, with an NH_2_- group for immobilization on the SPR sensor chips. Then, we used recombinant His-PP2AA1 and His-PP2AA3 proteins and measured their binding affinity to immobilized SA (Figures S5C–S5F). Indeed, we detected a concentration-dependent binding of His-PP2AA1 to immobilized SA. Plotted with the steady-state binding with different concentrations of the protein, we obtained a K_D_ of 3.623 μM (Figures 6D and S7D). Performing the same experiment for His-PP2AA3, we also detected binding with an even smaller K_D_ value of 1.916 μM (Figures 6E and S7E). In a different, single-cycle SPR experimental setup, including 0.1% BSA in the SPR flow to prevent unspecific binding, a similar K_D_ value (2.374 μM) for PP2AA1 was obtained (Figures S7F and S7G).

Thus, all these methodically distinct approaches have confirmed a direct binding of SA to A subunits of PP2A at concentrations well matching the SA physiological activity. The binding of SA to PP2AAs is in line with observations on SA regulating PP2A activity, downstream PIN2 (de)phosphorylation, and auxin-mediated root development.

## Discussion

Balancing allocation of resources between growth and defense against pathogens is a common challenge in multicellular organisms [57]. It has been long proposed that, except for the canonical roles as stress hormones, both SA and jasmonic acid (JA) also regulate plant growth and development [12, 15]. Meanwhile, another phytohormone, auxin, well recognized as an essential signaling molecule for growth and development and seemingly involved in almost every aspect of plant life, was proposed to also participate in plant defense against pathogens [11, 15, 58]. Here, we revealed a dual role for the plant hormone SA, which by two parallel perception and signaling mechanisms concomitantly activates immunity and represses growth.

**Figure 7:**
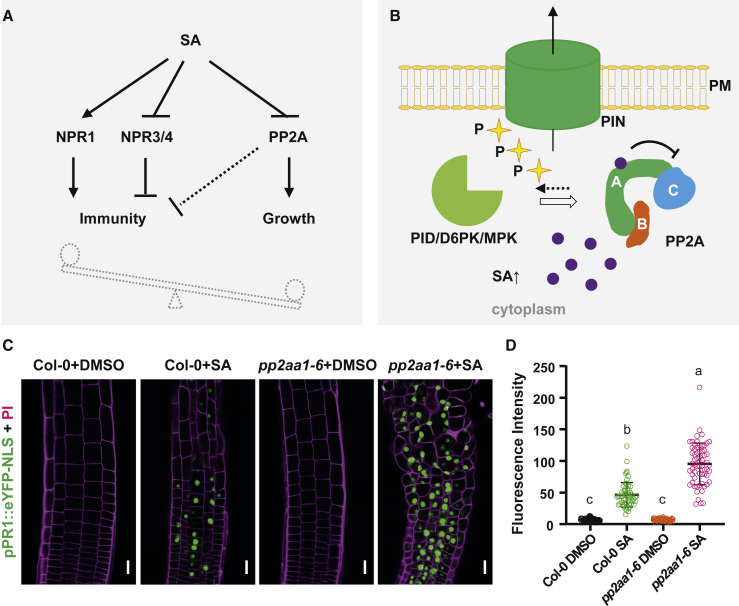
Model for the Parallel SA Action in Immunity and Growth Regulation (A) SA plays a key role in the growth-immunity transition following pathogen attack: on one hand, SA activates the immune response, through stimulating NPR1 and repressing NPR3/4, all together increasing the expression of downstream defense genes; on the other hand, SA inhibits growth via suppressing PP2A activity and the subsequent dephosphorylation of substrates. (B) The auxin efflux carrier PIN2 is phosphorylated by different kinases, including PINOID/WAGs, D6PK/D6PKLs, and MAPKs, and dephosphorylated by PP2A. Following pathogen attack, the SA levels increase. SA binds to the A subunits of PP2A and thereafter represses its dephosphorylation activity toward PIN proteins, which leads to hyperphosphorylation of PIN, thereby a decrease in PIN activity ultimately resulting in a decrease in auxin export and attenuation of growth. (C and D) Induced stronger expression of *pPR1::eYFP-NLS* by SA was detected in *pp2aa1-6*. (C) *pPR1::eYFP-NLS* seedlings were constantly grown on plates with DMSO or 40 μM SA for 5 days from germination and were then imaged by CLSM. Scale bars, 10 μm. (D) For quantification, the average GFP florescence of 5–10 representative cells from 10 seedlings for each treatment was measured by Fiji. The data points were showed as dot plots, and lines indicate mean ± SD. Different letters represent significant difference; p < 0.05; by one-way ANOVA with a Tukey multiple comparison test.

SA is a well-established defense signal of plants; its levels rapidly rise following pathogen attack, and it acts via the NPR-type receptors on transcription of defense genes (Figure 7A) [1]. Here, we identify a parallel signaling pathway that leads to regulation of growth. Both *in vivo* and *in vitro* experiments show that SA specifically binds to the A subunit of the PP2A complex and inhibits its activity. PP2A is a protein phosphatase important for many cellular processes through dephosphorylating various protein substrates [43, 45, 48]. Prominent among its substrates are PIN auxin transporters that play key roles in many developmental processes, and multiple aspects of PIN activity, localization, and subcellular dynamics are mediated by different phosphorylation states [27, 39]. In line with our observation that SA inhibits PP2A activity, increased SA levels lead to an increase in PIN phosphorylation and thus to a change in subcellular PIN distribution and decrease in auxin export activity (Figure 7B). This leads to attenuation of auxin-mediated growth as manifested by a decrease in primary root elongation, inhibition of gravitropic response, and repression of lateral root organogenesis. Identification of SA as direct regulator of PP2A highlights a role for this phosphatase complex as a molecular hub for the trade-off between immune response and growth. It is noteworthy that SA does not completely inhibit the PP2A activity, perhaps because PP2AAs are solely the scaffold proteins for the PP2A holoenzyme. This regulatory mode may present a mechanism to fine-tune PP2A activity under different conditions. Notably, we demonstrate that this hyperphosphorylation by PP2A inhibition leads to mislocalization of PIN2, suggesting more kinases, other than PID, involved in apical versus basal PIN targeting [43]. Phosphorylation by mitogen-activated protein kinase (MAPK) gives rise to a decreased PIN polarity and plasma membrane (PM) targeting [59]; thus, it would be interesting to investigate whether PP2A also antagonizes with MAPK in directing PIN localization.

Our previous study revealed that SA interferes with the internalization of PIN proteins, which depends on the clathrin-mediated endocytosis pathway [14]. It has been also reported that *pp2a* mutants, including *pp2aa1*, show decreased PIN internalization [60, 61]. Our proposed SA-PP2A model further clarifies the molecular mechanism underlying the SA effect on PIN trafficking [14]. A recent study shows that SA has an impact on the root meristem patterning via auxin distribution through both upregulating auxin biosynthesis and interfering with transport [30]. Though elevated auxin levels do not typically lead to agravitropic root growth and therefore are likely a result of a regulatory feedback from the blocked auxin transport, it would still be interesting to test whether PP2A is also involved in this SA effect. Likewise, the observed developmental abnormalities in SA-treated root columella cells [30] were also reported in PP2A mutants [48], further supporting our hypothesis that this SA-PP2A pathway plays more roles in plant growth and development. Here, this study focuses on the SA action on root development, especially PIN2-mediated gravitropism. It is likely that more PP2A substrates, other PIN proteins, or even non-PIN substrates are also involved in these effects.

Previous studies uncovered that plant pathogens interfere with the auxin pathway at the level of the signaling. For example, flagellin of pathogen can induce a microRNA (miRNA) to negatively regulate the expression of auxin receptors, TRANSPORT INHIBITOR RESISTANT1 (TIR1)/ AUXIN SINGNALING F BOX (AFB) [58]. Moreover, SA also stabilizes the negative regulators of auxin signaling pathway, AUXIN/IAA (AUX/IAA) [8], or interferes with auxin biosynthesis [11]. Notably, the *npr1* mutation suppresses the immune response, but not the growth attenuation phenotype of *snc2-1D*, which shows constitutive defense response [62]. Recently, a gain-of-function mutation of *NPR4*, *npr4-4D*, was identified to work together with *npr1-1* and additively to regulate immune response as well as the growth pathway [7]. In view of these observations, we conclude that SA regulates plant growth and development through multiple mechanisms, many of which involve auxin. Generally, it remains unclear whether these other SA effects are mediated by the canonical, NPR1-mediated pathway or require here identified SA-PP2A signaling module.

Notably, by investigating the NPR1-mediated immune response with *pPR1::eYFP-NLS*, we found that *pp2aa1* mutation leads to an increased SA sensitivity (Figures 7C and 7D). It has been reported that bacterial type-III effector proteins could target PP2A to facilitate infection and that multiple *pp2a* loss-of-function mutants, including *pp2aa1*, exhibit elevated response to pathogen attack [63]. Together with our findings, we hypothesize that PP2A, as an essential regulator for multiple pathways, might play a central role in coordinating plant immune response with attenuation of growth and development.

Previous studies demonstrated that NPR1/NPR3/NPR4 are genuine SA receptors, mediating the downstream transcriptional response. NPR proteins share sequence similarity with the mammalian master regulator in the inflammatory response, nuclear factor κB (NF-κB), and specifically its subunit, inhibitor protein I-κB (IκB) [3, 4]. IκB is phosphorylated by an IκB kinase (IKK) complex, whose activity is directly inhibited by salicylates, the active breakdown compound of the common anti-inflammatory drug Aspirin (2-acetoxybenzoic acid), thus providing mechanism of their well-known anti-inflammatory effects [64]. These interesting analogies between plant and mammalian pathogen defense mechanisms, both at the sequence level of involved regulators as well as at structural level of the involved ligands, point to possible evolutionary conservation between these otherwise seemingly unrelated pathogen defense strategies. Given the fact that PP2A regulates the dephosphorylation of numerous substrates, it would be interesting to investigate whether the SA-PP2A signaling module is a part of this evolutionary conserved mechanism and also regulates the NPR-mediated immunity in plants or NF-κB-controlled inflammatory response in mammals.

## STAR★Methods

### Key Resources Table

**Table:** 

REAGENT or RESOURCE	SOURCE	IDENTIFIER
**Antibodies**		

Rabbit anti-PIN1	[37]	N/A
Rabbit anti-PIN2	[36]	N/A
Mouse anti-His-tag monoclonal Antibody	Agrisera	Cat# AS11 1771
Mouse anti-myc-tag monoclonal Antibody, clone 4A6	Millipore (Merck)	Cat# 05-724
Phospho-PP2A alpha (Tyr307) Polyclonal Antibody	Thermo Scientific	Cat# PA5-36874; RRID:AB_2553794
Anti-GFP-HRP	Miltenyi Biotec	Cat# 130-091-833; RRID:AB_247003
Monoclonal anti-GFP antibody produced in mouse	Sigma	Cat# G6539; RRID:AB_259941
Anti-PIP2;1	[65]	N/A

**Bacterial and Virus Strains**		

*Escherichia coli* DH5α	Lab stock	N/A
*E.coli* BL21 (DE3)	New England Biolabs	Cat# C2527H
*Agrobacterium tumefaciens* GV3101	Lab stock	N/A
*Pseudomonas syringae pv. tomato* DC3000	Armin Djamei lab	N/A

**Chemicals, Peptides, and Recombinant Proteins**		

brefeldin A	Sigma	Cat# B7651
Propidium Iodide	Sigma	Cat# P3566
PBS Buffer 10 × (1000 mL)	GE Healthcare	Cat# BR100672
PhosSTOP	Sigma/Roche	Cat# 4906837001
cOmplete protease inhibitor cocktail	Sigma/Roche	Cat# 4693124001
Benzoic acid (BA)	Sigma	Cat# 242381
Salicylic Acid (SA)	Sigma	Cat# 247588
3-Hydroxybenzoic acid (3-OH-BA)	Sigma	Cat# H20008
4-Hydroxybenzoic acid (4-OH-BA)	Sigma	Cat# H20059
N-(1-Naphthyl)phthalamidic acid	Sigma	Cat# N12507
2,3,5-Triiodobenzoic acid (TIBA)	Sigma	Cat# T5910
[3H]-IAA (([5-^3^H]-Indole-3-acetic acid)	American Radiolabeled Chemicals	Cat# ART 0340
[3H]- NAA ([4-^3^H]-1-Naphthylacetic acid)	American Radiolabeled Chemicals	Cat# ART 0610
[3H]-2,4-D (([5-^3^H]-2,4-Dichlorophenoxy acetic acid)	American Radiolabeled Chemicals	Cat# ART 0559
Cantharidin	Sigma	Cat# C7632
Imidazole	Sigma	Cat# I5513
FastDigest Hin1II	Thermo Fisher Scientific	Cat# FD1834
FastDigest EcoRI	Thermo Fisher Scientific	Cat# FD0274
FastDigest XhoI	Thermo Fisher Scientific	Cat# FD0694
FastDigest BamHI	Thermo Fisher Scientific	Cat# FD0054
FastDigest SalI	Thermo Fisher Scientific	Cat# FD0644
T4 DNA Ligase Buffer	Thermo Fisher Scientific	Cat# 46300-018
T4 DNA Ligase (1 U/μL)	Thermo Fisher Scientific	Cat# 15224-017
GeneJET Plasmid Miniprep Kit	Thermo Fisher Scientific	Cat# K0503
GeneJET Gel extraction kit	Thermo Fisher Scientific	Cat# K0692
BSA (Bovine Serum Albumin)	Sigma	Cat# A2153
His-PP2AA1	This study	N/A
His-PP2AA3	This study	N/A
His-PIN2HL	This study	N/A

**Critical Commercial Assays**		

Bio-Safe Coomassie Stain #1610786	Bio-Rad	Cat# 1610786
Non-Radioactive Phosphatase Assay Systems	Promega	Cat# V2460
HisPur Ni-NTA Resin	Thermo Fisher Scientific	Cat# 88222
γ-[^32^P]-ATP	PerkinElmer	Cat# NEG502A001MC

**Experimental Models: Cell Lines**		

*Nicotiana tabacum* L., cv. Bright Yellow-2 (BY-2)	N/A	N/A

**Experimental Models: Organisms/Strains**		

*Arabidopsis thaliana* Col-0	N/A	N/A
*A. thaliana* Ws-4	NASC	N5390
*A. thaliana eir1-4* (*pin2-T*)	[36]	SALK_091142
*A. thaliana aux1-T (aux1)*	[66]	SALK_020355
*A. thaliana pAUX1::AUX1-YFP*	[67]	N/A
*A. thaliana pPR1::eYFP-NLS*	[24]	N/A
*A. thaliana npr1-1*	[2]	N/A
*A. thaliana npr3-1 npr4-3*	[68]	N/A
*A. thaliana npr1-1 npr3-1 npr4-3*	[68]	N/A
*A. thaliana cpr6*	[23]	N/A
*A. thaliana sid2-3*	[69]	SALK_042603
*A. thaliana rcn1-1 (rcn1, pp2aa1-1)*	[42]	N/A
*A. thaliana rcn1-6 (pp2aa1-6)*	[49]	SALK_059903
*A. thaliana pp2aa2-2*	[45]	SALK_037095
*A. thaliana pp2aa2-3*	[45]	SALK_017541
*A. thaliana pp2aa3-2*	[45]	SALK_099550
*A. thaliana pp2aa1 pp2aa2-3*	[45]	N/A
*A. thaliana pp2aa1 pp2aa3-1*	[45]	N/A
*A. thaliana pPIN2::PIN2-GFP*	[70]	N/A
*A. thaliana pPP2AA1::PP2AA1-GFP*	[45]	N/A
*A. thaliana DR5v2*	[28]	N/A
*A. thaliana eir1-4 DR5v2*	This study	N/A
*A. thaliana pPR1::eYFP-NLS*	[24]	N/A
*A. thaliana pp2aa1-6 pPR1::eYFP-NLS*	This study	N/A
*A. thaliana 35S::4 × myc-PP2AA1*	This study	N/A
*A. thaliana pp2aa1-6 cpr6*	This study	N/A

**Oligonucleotides**		

Primers used in this study, see Table S1	This study	N/A

**Recombinant DNA**		

Plasmid pET28a-PP2AA1	This study	N/A
Plasmid pET28a-PP2AA3	This study	N/A
Plasmid pET28a-PIN2HL	This study	N/A
Plasmid pEGAD-35S::4 × myc-PP2AA1	This study	N/A

**Software and Algorithms**		

*Arabidopsis* Information Resource (TAIR)	http://www.arabidopsis.org/	N/A
ImageJ	https://imagej.nih.gov/ij/	NIH
Fiji	https://fiji.sc/	N/A
ZEN	https://www.zeiss.com/microscopy/int/products/microscope-software/zen-lite.html	ZEISS
DNA MAN	https://www.lynnon.com/	N/A
ChemSketch	https://www.acdlabs.com/resources/freeware/chemsketch/	N/A

### Lead Contact and Materials Availability

Requests for resources and reagents such as plasmids, compounds, mutant and transgenic lines should be directed to and will be fulfilled by the Lead Contact, Jiří Friml (jiri.friml@ist.ac.at).

### Experimental Model and Subject Details

#### Plant Materials and Growth Conditions

*Arabidopsis thaliana* (L.) mutants or transgenic lines are in Columbia-0 (Col-0) background if not particularly mentioned. The mutants and marker lines *pPIN2::PIN2-GFP* in *eir1-1* [70], *pAUX1::AUX1-YFP* [67], *aux1-T* [66], *eir1-4* (*pin2-T*) [36], *npr1-1* [2, 3], *sid2-3* (*sid2*) [69], *npr3-1 npr4-3* [68], *npr3-2 npr4-2* [68], *npr1-1 npr3-1 npr4-3* [68], *cpr1* [71], *cpr5* [72], *cpr6* [23], *rcn1-1* (*pp2aa1-1*, in Ws) [42], *rcn1-6* (*pp2aa1-6*) [49], *pp2aa2-2* [45], *pp2aa2-3* [45], *pp2aa3-2* [45], *pp2aa1,a2* (*pp2aa1, pp2aa2-3*) [45], *pp2aa1,a3* [45], *pp2aa2,a3* [45], *pPP2AA1::PP2AA1-GFP in Col-0* [45] and *DR5v2* [28] were published previously. The detailed information of plant lines, including mutants and marker lines, used in this study is listed in Key Resources Table. The primers used for genotyping the mutants were listed in Table S1.

For physiological experiments, surface-sterilized seeds were sown on Murashige and Skoog (1/2 MS) medium, supplemented with 1% sucrose, 0.8% phytoagar (pH 5.9), stratified at 4°C for 3 days (d), and then grown vertically in a growth chamber at 21°C with a 16-h-light/8-h-dark photoperiod.

### Method Details

#### Pseudomonas syringae treatment of *Arabidopsis* seedlings

*P. syringae* treatment was performed as reported previously [73]. A single colony of *P. syringae pv. tomato DC3000* (kind gift from Dr. Armin Djamei, IPK- Gatersleben) was cultured in 20 mL King’s B (KB) liquid media overnight, to get OD_600_ between 0.4 and 0.6. The DC3000 cells were collected by spinning down at 1600 g, and were then resuspended in infection buffer (0.025% Silwet L-77, and 10 mM MgCl_2_). The concentration was adjusted to OD_600_ = 0.01 ( = ∼5 × 10^6^ CFU/mL) for treatment. The DC3000 suspension was dispensed into the plates with 5-day-old *pPR1::eYFP-NLS* seedlings and incubated for 3 min at 25°C. Afterward, the suspension was decanted, and seedlings were grown for another 2 days before imaging.

#### Pharmacological treatments

For long-term growth experiments, seeds were sown on MS plates containing indicated chemicals, including benzoic acid (Sigma, 242381), SA (Sigma, 247588), 3-OH-BA (Sigma, H20008), 4-OH-BA (Sigma, H20059), cantharidin (Sigma, C7632), NPA (Sigma, N12507), and TIBA (Sigma, T5910). After 3-d stratification at 4°C, they were moved to grow in a growth chamber as mentioned in the “Plant material and growth conditions” section, for 7 d or 10 d.

For short-term treatment, 4-d-old seedlings were incubated in liquid MS medium containing indicated chemicals for a certain time course as described in the Figure Legends. Detailed information of all chemicals used in this study is listed in Key Resources Table.

#### Free SA measurement by liquid chromatography-tandem mass spectrometry (LC-MS/MS)

Free SA contents was measured by LC-MS/MS as previously reported [74]. Approximately 10 mg fresh weight (FW) of roots from Col-0, *sid2-3*, and *cpr6* were collected and frozen in liquid nitrogen for LC-MS/MS. SA contents were calculated by the whole amount divided by the fresh weight (pmol/g FW).

#### Auxin transport in hypocotyls and tobacco BY-2 cells

The basipetal (rootward) transport assay of [^3^H]-IAA in etiolated hypocotyls was performed according to a previous report [75], with a few modifications. 6-day-old etiolated Col-0 seedlings were placed on MS plates containing indicated chemicals, with 15 seedlings as one biological replicate, and 3 replicates per treatment. The [^3^H]-IAA (PerkinElmer, ART-0340) droplets were prepared in MS medium with 1.25% agar and 500 μM [^3^H]-IAA (1.45 μL in 10 mL), supplemented with same concentration of the chemicals as in the respective plate. The seedlings were decapitated and then covered with a [^3^H]-IAA droplet at the shootward end. After incubation for 6 hours in the dark, the lower part of the hypocotyls was cut and collected and were then ground completely in liquid nitrogen and homogenized in 1 mL scintillation solution (PerkinElmer, 6013199). The samples were incubated overnight to allow the radioactivity to evenly diffuse into the whole volume of the scintillation cocktail. Finally the radioactivity was measured with a scintillation counter (Hidex 300XL), with each sample counted for 100 s, 3 times. 3 samples with only the scintillation solution were used as background controls.

The transport of [^3^H]-NAA, [^3^H]-2,4-D, and [^3^H]-BA in tobacco BY-2 cells was performed as published previously [32].

#### Imaging with Confocal Laser Scanning Microscopy (CLSM)

Fluorescence imaging was performed using a Zeiss LSM800 confocal laser scanning microscope (CLSM) with a GaAsP detector (Zeiss, Germany). The manufacturer’s default settings (smart mode) were used for imaging GFP (excitation, 488 nm; emission, 495-545 nm)-, and tdTomato (excitation 561 nm; emission, 571-630 nm)-tagged proteins respectively. To image FM4-64-stained cells, a laser line of 543 nm was used for excitation, and an emission light with a wavelength of 600-700 nm was collected. For PI staining, excitation of 561 nm was used and emission signal was collected using a filter of 580- 680 nm. All images were recorded in 8 bit depth, 2 × line averaging. The images were analyzed and visualized with Fiji program [76].

#### Image analysis and morphological analysis

For root length measurement, photos were taken with a scanner (Epson Perfection V800 Photo) and then the root length was measured with ImageJ. The representative photos were taken by a camera (Sony A600 with a macro lens, 30mm/F3.5).

#### Molecular cloning

For pET28a-PIN2HL, pET28a-PP2AA1 and pET28a-PP2AA3 constructs, coding regions of *PIN2HL*, *PP2AA1* (primers PP2AA1-1/ PP2AA1-2) and *PP2AA3* (primers PP2AA3-1/ PP2AA3-2) were amplified and subcloned into vector pET28a (Novagen) with EcoRI/SalI, EcoRI/*Xho*I, and EcoRI/*Xho*I respectively.

All the plasmids were identified by PCR and confirmed by sequencing (LGC). The primers used were listed in Table S1.

#### PP2A activity assay

The total PP2A activity assay was performed as previously reported with a Ser/Thr protein phosphatase assay kit (Promega, V2460) [48]. Approximately 1g of 7-d-old seedlings were ground in liquid nitrogen. Phosphatase storage buffer (250 mM imidazole, 1 mM EGTA, 0.1% β-mercaptoethanol, and 0.5mg/ml BSA, pH7.2) was added (1/2, volume/weight, hereafter short as v/w) to the frozen tissues and centrifuged to remove cell debris. Endogenous free phosphate was removed with the supplied Sephadex G-25 columns. PP2A phosphatase activity was measured using a molybdate dye-based phosphatase assay kit (Promega, V2460). The reactions were incubated at 37°C for 30 min before being terminated by the molybdate dye and additive mixture. The transparent 96-well plate was read on a Biotek Synergy H1 plate reader at 25°C at 600 nm, with 4 reads per well. The experiment was performed in three independent biological replicates for each treatment.

#### Protein extraction and immunoblot

To examine the expression level of myc-PP2AA1 in the *35S::myc-PP2AA1* overexpression line, or the phosphorylation level at Tyr307 (Y307) of PP2A C subunits, 100 mg of 7-d-old Col-0 seedlings were frozen in liquid nitrogen, ground totally, and homogenized in plant extraction buffer (20 mM Tris-HCl, pH 7.5, 150 mM NaCl, 0.5% Tween-20, 1 mM EDTA (ethylenediaminetetraacetic acid), 1 mM DTT (1,4-dithiothreitol)) containing a protease inhibitor cocktail (cOmplete, Roche). After addition of an equal volume of 3 × SDS (sodium dodecyl sulfate) loading buffer, the samples were boiled for 5 min, fractionated by 10% SDS-PAGE (sodium dodecyl sulfate-polyacrylamide gel electrophoresis) and transferred to a PVDF membrane by wet blotting. The membrane were incubated with a mouse anti-myc antibody (Millipore) or a mouse pY307-PP2Ac antibody (Millipore) and then with a bovine anti-mouse IgG HRP (horseradish peroxidase)-conjugated secondary antibody (GE Healthcare). HRP activity was detected by the Supersignal Western Detection Reagents (Thermo Scientific) and imaged with a GE Healthcare Amersham 600RGB system.

#### PIN2 phosphorylation assays

Roots from Col-0 and *pp2aa1-6* were treated with 40 μM SA or DMSO for 15 min, 1 h and 2 h. Untreated roots were also collected at time zero from Col-0, *pp2aa1-6* and *eir1-4* respectively. Protein extraction was performed as previously [41], with modifications for preserving phosphorylation status. The extraction buffer (EB) was: 50 mM Na_2_HPO_4_ (pH 7.4), 25% w/w sucrose, 7.5% glycerol, 20 mM betaglycerolphosphate, 5 mM Na_2_MoO_4_, 50 mM NaF, 0.1% casein, 10 mM EDTA (pH 8), 5 mM EGTA (pH 8), 20 mM borate/10 mM Tris-HCl (pH 8.2), 1 mM Na_3_VO_4_, 10 nM okadaic acid, 1 × PhosStop (Roche). Protease inhibitors (1 mM PMSF (phenylmethanesulfonyl fluoride), 1 mM Pefabloc-SC, 2 μg/mL E64, 0.7 μg/mL pepstatin A, 1 μg/mL aprotinin, and 1 μg/mL leupeptin) and insoluble PVPP (polyvinylpolypyrrolidone) were used. Samples were milled in liquid N_2_, extracted with 4 volumes of EB, transferred to PVPP and spun at 500 g (2 min, 4°C). The supernatant was cleared again at 400 g (3 min, 4°C). The supernatant was saved as a total protein fraction, or diluted with 2 volumes of water and spun at 21, 000 g (20 min, 4°C) or 55, 000 g (10 min, 4°C) to obtain a membrane fraction pellet. All samples were solubilized with 0.5% SDS plus 20 mM DTE (Dithioerythritol), and precipitated with chloroform/methanol. Samples (corresponding to 2 or 3 mg original root weight) were denatured by heated only at 50°C to avoid aggregation, and were separated by SDS-PAGE and blotted. Blots were Ponceau stained to confirm loading, probed with rabbit anti-PIN2 [36], stripped and reprobed with anti-PIN1 [37] or anti-PIP2;1 [65]. HRP activity was detected by the Supersignal Western Detection Reagents (Thermo Scientific) and imaged with Biorad XRS Chemidoc or conventional film.

#### PIN2-HL phosphorylation assay with [γ-^32^P]-ATP

The phosphorylation assay of PIN2-HL with [γ-^32^P]-ATP was performed as previously described [43], with a few modifications. Roots from Col-0 and *pp2aa1-6* (approximately 100 mg) were treated with 40 μM SA or DMSO for 1 h, and harvested for protein extraction. The samples were ground in liquid nitrogen and homogenized in 100 μL protein extraction buffer (20 mM Tris-HCl, pH 7.5, 150 mM NaCl, 0.5% Tween-20, 1 mM DTT, cOmplete protease inhibitor cocktail). 20 μL (∼10 μg) recombinant His-PIN2HL protein was added with 4 μL plant extract, and then the reaction was initiated by adding 10 mM MgCl_2_ and 2 μL (20 μCi) [γ-^32^P]-ATP (NEG502A001MC, Perkin-Elmer). After incubation at 25°C for 1h, the reaction was terminated by adding 10 μL SDS loading buffer. The protein samples were separated by SDS-PAGE. The gel was rinsed with deionized H_2_O, covered with a thin transparent plastic membrane, and developed with a phosphor plate overnight. The phosphor plate was finally scanned with a Fujifilm FLA 3000 plus DAGE system.

#### Drug Affinity Responsive Target Stability (DARTS) assay

The DARTS assay to test the binding of SA to PP2AA1-GFP was performed as previously reported [77, 78]. *pPP2AA1::PP2AA1-GFP* seedlings (7d) were used for total protein extraction. After harvesting, the samples were ground in liquid nitrogen, resuspended in protein extraction buffer (25 mM Tris-HCl, pH 7.5; 150 mM NaCl; 0.1% IGEPAL CA-630, Roche cOmplete protease inhibitor cocktail, EDTA free) with a 1:2 (w/v) ratio, and spun down to discard the cell debris. After quantifying the protein concentration (Quick Start Bradford Reagent, Bio-Rad), the cell lysate was aliquoted and incubated with 0, 50 μM or 500 μM SA respectively for 30 min at 25°C, mixing at a low speed. The treated extracts were further aliquoted, and mixed with different concentrations of Pronase (Roche) in Pronase buffer (25 mM Tris-HCl, pH 7.5; 150 mM NaCl). After incubation at 25°C for 30 min, the proteolytic digestion was terminated by adding protease inhibitor cocktail (cOmplete, Roche) and the samples were kept on ice for 10 min. The protein samples were then analyzed by western blot. PP2AA1-GFP was detected by an anti-GFP antibody (JL8, Clontech, 1:2000). HRP activity was detected by the Supersignal Western Detection Reagents (Thermo Scientific) and imaged with a GE Healthcare Amersham 600RGB system.

#### Recombinant protein expression and purification

Recombinant proteins were expressed in the *E. coli* strain BL21 (DE3) with induction by 0.5 mM IPTG (Isopropyl β-D-1-Thiogalactopyranoside, 16°C, 12 h) and then purified using Ni-NTA His binding resin (Thermo Scientific) according to the manufacturer’s manual. The eluted samples were then purified with size exclusion chromatography, with a Superdex 200 increase column, on an ÄKTA pure chromatography system (GE Healthcare). Fractions were collected by 500 μL, and then analyzed by SDS-PAGE, followed by Coomassie brilliant blue (CBB, Bio-Safe Coomassie Stain #1610786 from BioRad) staining to check the protein quality.

#### Differential Scanning Calorimetry (DSC) analysis

The DSC analysis was performed with a MicroCal PEAQ-DSC Automated instrument (Malvern Panalytical). 5 μM PP2AA1 in 1 × PBS, with or without 50 μM SA, were heated from 25°C to 85°C at a heating rate of 1°C /min, cooled *in situ* and heated again under the same conditions. Data was obtained and analyzed with the provided program.

#### Chemical synthesis of SA derivatives

##### General information

All starting materials were used as received from commercial sources (Sigma-Aldrich, Merck, and Lach-Ner) without further purification. 2-(6-bromohexyl)isoindoline-1,3-dione was prepared using published procedure. THF [79] was distilled under argon from sodium benzophenone ketyl. All reactions were performed in round-bottom flasks fitted with rubber septa using the standard laboratory techniques. Reactions sensitive to air and/or moisture were performed under a positive pressure of argon. Analytical thin-layer chromatography (TLC) was performed using aluminum plates pre-coated with silica gel (silica gel 60 F^254^). TLC plates were visualized by exposure to ultraviolet light and then were stained by submersion in basic potassium permanganate solution or in ethanolic phosphomolybdic acid solution followed by brief heating. Column chromatography was performed on silica gel 60 (40-63 μm). Melting points (mp) were tested on a capillary melting point apparatus. ^1^H NMR and ^13^C NMR spectra were recorded on 500 and 125 MHz in CDCl_3_, CD_3_OD, acetone-*d*_*6*_ and DMSO-*d*_*6*_; chemical shifts (δ ppm) and coupling constants (Hz) of ^1^H NMR are reported in a standard fashion with relative to the remaining CHCl_3_ present in CDCl_3_ (δH = 7.27 ppm), central line of pentet in CHD_2_OD present in CD_3_OD (δH = 3.31 ppm), central line of pentet in CHD_2_C(O)CD_3_ present in acetone-*d*_*6*_ (δH = 2.05 ppm), and central line of pentet in CHD_2_SOCD_3_ present in DMSO-*d*_*6*_ (δH = 2.50 ppm). ^13^C NMR chemical shifts (δ ppm) are reported relative to CDCl_3_ (δC = 77.23 ppm, central line of triplet), CD_3_OD (δC = 49.0 ppm, central line of heptet), CD_3_C(O)CD_3_ (δC = 29.84 ppm, central line of heptet), and DMSO-*d*_*6*_ (δC = 39.52 ppm, central line of heptet). Proton coupling patterns are represented as singlet (s), doublet (d), doublet of doublet (dd), triplet (t), triplet of triplet (tt), pentet (p), and multiplet (m). HRMS data were obtained using quadrupole/ion trap mass analyzer. Analysis and assignments were made by comparison with literature spectroscopic data or using 2D-COSY, HSQC, HMBC, 2D-NOESY and 1D-NOEdiff experiments. Purity of final compounds was determined using the following protocol: Compound (1 mg) was dissolved in 1 mL of 1% methanol and injected (10 μL) onto a reverse-phased column (Symmetry C18, 5 μm, 150 mm × 2.1 mm; Waters, Milford, MA, USA) incubated at 25°C. Solvent (A) consisted of 15 mM ammonium formate adjusted to pH 4.0. Solvent (B) consisted of methanol. At flow-rate of 200 μL/min, following binary gradient was used: 0 min, 10% B; 0-24 min. linear gradient to 90% B; 25-34 min. isocratic elution of 90% B; 35-45 min. linear gradient to 10% B. The effluent was introduced then to PDA detector (scanning range 210-700 nm with 1.2 nm resolution) and an electrospray source (source temperature 120°C, desolvation temperature 300°C, capillary voltage 3 kV, cone voltage 20 V). Nitrogen was used as well as cone gas (50 L/h) and desolvation gas (500 L/h). Data acquisition was performed in the full scan mode (50-1000 Da), scan time of 0.5 s. and collision energy of 6 V. Analyses were performed in positive mode (ESI^+^) or in negative mode (ESI^-^), therefore data were collected as quasi-molecular ions of [M+H]^+^ and [M-H]^-^, respectively.

##### C-10 (5-(allyloxy)-2-hydroxybenzoic acid)

Successively, K_2_CO_3_ (1.23 g, 8.93 mmol, 1.5 equiv) and allyl bromide (0.643 mL, 7.4 mmol, 1.25 equiv) were added to a solution of methyl 2,5-dihydroxybenzoate (1.0 g, 6.0 mmol, 1.0 equiv) in dry acetone (60 mL) and the resulting mixture was heated up to 60°C. After 5h at 60°C, the reaction mixture was cooled to 25°C (room temperature) and diluted with H_2_O (50 mL). The whole mixture was extracted with CH_2_Cl_2_ (3 × 75 mL). Organic layers were combined and washed with brine (50 mL), dried over MgSO_4_, filtered and volatiles were removed under reduced pressure. The residue was purified by flash column chromatography (SiO_2_; hexane:EtOAc = 20:1 - > 10:1) and yielded 5-O-allylated ester (0.719 g, 58%). ^1^H NMR (500 MHz, CDCl_3_) δ (ppm): 3.95 (s, 3H), 4.52 (dt, *J* = 5.2, 1.8 Hz, 2H), 5.32 (dd, *J* = 10.5, 1.6 Hz, 1H), 5.43 (dd, *J* = 17.2, 1.7 Hz, 1H), 6.06 (ddt, *J* = 17.6, 10.5, 5.3 Hz, 1H), 6.92 (d, *J* = 9.2 Hz, 1H), 7.11 (dd, *J* = 9.2, 3.2 Hz, 1H), 7.32 (d, *J* = 3.2 Hz, 1H), 10.39 (s, 1H); 13C NMR (126 MHz, CDCl_3_) δ (ppm): 52.3, 69.5, 111.7, 113.1, 117.8, 118.4, 124.6, 133.1, 150.8, 156.1, 170.2; MS (ESI^+^), *m/z* (%): 209 [M+H]^+^ (100); HRMS (ESI^+^) *calcd.* for C_11_H_13_O_4_ [M+H]^+^: 209.0808, found 209.0808. 5-O-allylated ester (0.5 g, 2.4 mmol, 1.0 equiv) was dissolved in dry THF (24 mL) at 25°C. Potassium trimethylsilanolate (TMSOK, 0.924 g, 7.2 mmol, 3.0 equiv) was added and the resulting mixture was stirred at 25°C for 24 h. After this period of time, pH of the reaction mixture was adjusted to 2 with help of 10% aq. HCl. Organic solvents were removed under reduced pressure and additional H_2_O (20 mL) was added. The whole mixture was extracted with CH_2_Cl_2_ (2 × 50 mL) and combined organic layers were washed with brine (30 mL), dried over MgSO_4_ and evaporated to dryness under reduced pressure. The residue was purified by column chromatography (SiO_2_; hexan:EtOAc:AcOH = 2:1:0.1 - > 1:1:0.1) to yield the desired compound C-10 (364 mg, 78%). ^1^H NMR (500 MHz, CDCl_3_) δ (ppm): 4.53 (dt, *J* = 5.3, 1.7 Hz, 2H), 5.32 (dd, *J* = 10.6, 1.5 Hz, 1H), 5.43 (dd, *J* = 17.4, 1.6 Hz, 1H), 6.06 (ddt, *J* = 17.5, 10.6, 5.4 Hz, 1H), 6.96 (d, *J* = 9.2 Hz, 1H), 7.19 (dd, *J* = 9.0, 3.1 Hz, 1H), 7.39 (d, *J* = 3.3 Hz, 1H), 10.03 (s, 1H); ^13^C NMR (126 MHz, CDCl_3_) δ (ppm): 69.8, 110.9, 113.8, 118.2, 119.1, 126.6, 133.2, 151.4, 157.1, 174.7; MS (ESI^+^), *m/z* (%): 195 [M+H]^+^; HRMS (ESI^+^) *calcd.* for C_10_H_11_O_4_ [M+H]^+^: 195.0652, found 195.0651.

##### SA-1 (5-((6-aminohexyl)oxy)-2-hydroxybenzaldehyde hydrochlorid)

SA-3 (0.4 g, 1.09 mmol, 1.0 equiv) was dissolved in THF/H_2_O = 2:1 (9.0 mL) and the resulting solution was cooled to 0°C. A solution of HSO_3_(NH_2_) (0.211 g, 2.2 mmol, 2.0 equiv) in H_2_O (2.2 mL) followed by NaClO_2_ (0.108 g, 1.2 mmol, 1.1 equiv) in H_2_O (1.2 mL) was added, and the resulting mixture was stirred at 0°C for 2 h. H_2_O (20 mL) was added and the resulting solution was extracted with CH_2_Cl_2_ (3 × 50 mL). Organic layers were combined and washed with brine (25 mL), dried over Na_2_SO_4_, filtered and volatiles were removed under reduced pressure to yield carboxylic acid (0.343 g, 82%) sufficiently pure to be used in the next step. ^1^H NMR (500 MHz, acetone-*d*_6_) δ (ppm): 1.37 – 1.50 (m, 2H), 1.49 – 1.60 (m, 2H), 1.67 – 1.75 (m, 2H), 1.81 – 1.92 (m, 2H), 3.66 (t, J = 7.1 Hz, 2H), 4.20 (t, *J* = 6.6 Hz, 2H), 7.04 (dd, *J* = 8.9, 3.1 Hz, 1H), 7.11 (d, *J* = 9.0 Hz, 1H), 7.42 (d, *J* = 3.1 Hz, 1H), 7.92 (dd, *J* = 6.0, 3.3 Hz, 2H), 8.20 (dd, *J* = 6.0, 3.3 Hz, 2H); MS (ESI^+^), *m/z* (%): 384 [M+H]^+^; HRMS (ESI^+^) *calcd.* for C_21_H_21_NO_6_Na [M+Na]^+^: 406.1261, found 406.1262. Carboxylic acid (0.300 g, 0.78 mmol, 1.0 equiv) was dissolved in EtOH (8 mL) and hydrazine hydrate (0.076 mL, 1.56 mmol, 2.0 equiv) was added. The resulting mixture was stirred at 60°C for 6 h. White precipitate formed upon heating was filtered off and the filtrate was concentrated under reduced pressure to yield viscose oil. EtOH (10 mL) and H_2_O (10 mL) were added and the pH was adjusted to 2 with help of 2.0 M aq. HCl. Concentration of the resulting mixture under reduced pressure and subsequent co-evaporation of the residue with EtOH (2 × 10 mL) and toluene (2 × 15 mL) yielded desired compound SA-1 (0.052 g, 27%). Mp: > 190°C (dec.); ^1^H NMR (500 MHz, CD_3_OD) δ (ppm): 1.44 – 1.51 (m, 2H), 1.51 – 1.62 (m, 2H), 1.65 – 1.75 (m, 2H), 1.82 (ddt, *J* = 14.2, 7.9, 4.2 Hz, 2H), 2.94 (t, *J* = 7.6 Hz, 3H), 4.07 (t, *J* = 6.3 Hz, 2H), 6.96 (dd, *J* = 8.9, 3.0 Hz, 1H), 7.00 (d, *J* = 8.9 Hz, 1H), 7.24 (d, *J* = 3.0 Hz, 1H); ^13^C NMR (126 MHz, CD_3_OD) δ (ppm): 26.6, 27.1, 28.5, 30.1, 40.7, 70.8, 116.9, 118.1, 121.3, 122.3, 152.1, 157.8, 168.5; MS (ESI^+^), *m/z* (%): 254 [M-Cl]^+^; HRMS (ESI^+^) *calcd.* for C_13_H_20_NO_4_ [M-Cl]^+^: 254.1387, found 254.1388.

##### SA-2 (5-((5-(1,3-dioxoisoindolin-2-yl)pentyl)oxy)-2-hydroxybenzaldehyde)

2,5-dihydroxybenzaldehyde (0.5 g, 3.62 mmol, 1.0 equiv) was dissolved in dry DMF (36 mL) and K_2_CO_3_ (0.6 g, 4.3 mmol, 1.2 equiv) and 2-(6-bromohexyl)isoindoline-1,3-dione (1.07 g, 3.62 mmol, 1.0 equiv) were added. The resulting mixture was heated at 70°C for 4 h. All volatiles were removed under reduced pressure and the residue was dissolved in H_2_O (50 mL). The whole mixture was extracted with EtOAc (3 × 50 mL) and combined organic layers were washed with brine (25 mL), dried over Na_2_SO_4_, filtered and evaporated to dryness. The residue was purified by flash column chromatography (SiO_2_; hexane:EtOAc = 4:1- > 2:1) to yield SA-2 (0.627 g, 49%) as a yellowish viscose oil. ^1^H NMR (500 MHz, CDCl_3_) δ(ppm): 1.48 – 1.60 (m, 2H), 1.77 (p, *J* = 7.3 Hz, 2H), 1.82 – 1.93 (m, 2H), 3.73 (dd, *J* = 7.8, 6.5 Hz, 2H), 4.01 (t, *J* = 6.3 Hz, 2H), 6.85 (d, *J* = 9.0 Hz, 1H), 7.08 (dd, *J* = 8.9, 3.2 Hz, 1H), 7.28 (d, *J* = 3.1 Hz, 1H), 7.72 (dd, *J* = 5.5, 3.1 Hz, 2H), 7.85 (dd, *J* = 5.5, 3.1 Hz, 2H), 10.40 (s, 1H); ^13^C NMR (126 MHz, CDCl_3_) δ (ppm): 23.5, 28.4, 28.9, 37.9, 68.9, 113.5, 114.4, 123.4, 123.7, 125.4, 132.2, 134.2, 150.8, 155.8, 168.7, 189.9; MS (ESI^+^), *m/z* (%): 354 [M+H]^+^; HRMS (ESI^+^) *calcd.* for C_20_H_20_NO_5_ [M+H]^+^: 354.1336, found 354.1335.

##### SA-3 (5-((6-(1,3-dioxoisoindolin-2-yl)hexyl)oxy)-2-hydroxybenzaldehyde)

Using the same procedure as for SA-2 synthesis. The residue was purified by flash column chromatography (SiO_2_; hexane:EtOAc = 4:1- > 2:1) to yield SA-3 (1.04 g, 78%) as a white solid. Mp = 148-149°C; ^1^H NMR (500 MHz, CDCl_3_) δ (ppm): 1.41 (dd, *J* = 15.3, 7.9 Hz, 2H), 1.46 – 1.56 (m, 2H), 1.70 (dt, *J* = 15.0, 7.6 Hz, 2H), 1.74 – 1.84 (m, 2H), 3.69 (t, *J* = 7.3 Hz, 2H), 3.98 (t, *J* = 6.4 Hz, 2H), 6.83 (d, *J* = 9.2 Hz, 1H), 7.07 (dd, *J* = 8.9, 3.1 Hz, 1H), 7.28 (d, *J* = 3.3 Hz, 1H), 7.70 (dd, *J* = 5.3, 2.9 Hz, 2H), 7.82 (dd, *J* = 5.3, 2.9 Hz, 2H), 7.90 (s, 1H), 10.40 (s, 1H); ^13^C NMR (126 MHz, CDCl_3_) δ (ppm): 25.8, 26.7, 28.6, 29.2, 38.0, 69.1, 113.4, 114.4, 123.4, 123.7, 125.4, 132.2, 134.1, 150.9, 155.7, 168.7, 190.0; MS (ESI^+^), *m/z* (%): 368 [M+H]^+^; HRMS (ESI^+^) *calcd.* for C_21_H_22_NO_5_ [M+H]^+^: 368.1492, found 368.1492.

##### SA-f (5-((5-aminopentyl)oxy)-2-hydroxybenzoic acid)

Methyl 2,5-dihydroxybenzoate (4.1 g, 24.4 mmol, 1.0 equiv) was dissolved in acetone/H_2_O = 3.3:1 (190 mL) and K_2_CO_3_ (13.48 g, 98 mmol, 4 equiv) followed by 1,5-dibromopentane (10.0 mL, 73.4 mmol, 3.0 equiv) were added. The resulting mixture was refluxed for 4h, allowed to cool to 25°C and volatiles were removed under reduced pressure. Residue was extracted with CH_2_Cl_2_ (540 mL) and the organic layer was washed with H_2_O (220 mL), brine (150 mL), dried over MgSO_4_, filtered and evaporated to dryness yielding crude methyl 5-((5-bromopentyl)oxy)-2-hydroxybenzoate (16.5 g) as a brown oil. Crude ester was dissolved in acetone/H_2_O = 3.3:1 (190 mL) and NaN_3_ (7.9 g, 121.8 mmol, 5.0 equiv) was added. The resulting mixture was refluxed for 24 h before being allowed to cool to 25°C. Volatile solvents were removed under reduced pressure and the resulting mixture was extracted with CH_2_Cl_2_ (500 mL). Organic layer was washed with H_2_O (150 mL), brine (100 mL), dried over MgSO_4_, filtered and evaporated under reduced pressure. Resulting crude product was purified by flash column chromatography (SiO_2_; hexan:EtOAc = 20:1- > 10:1) and yielded the desired methyl 5-((5-azidopentyl)oxy)-2-hydroxybenzoate (6.8 g, 99%) as a colorless oil. ^1^H NMR (500 MHz, CDCl_3_) δ (ppm): 1.40 – 1.53 (m, 2H), 1.60 – 1.67 (m, 2H), 1.81 (dt, *J* = 14.6, 6.5 Hz, 2H), 3.30 (t, *J* = 6.8 Hz, 2H), 3.93 (t, *J* = 6.3 Hz, 2H), 3.96 (s, 3H), 6.92 (d, *J* = 9.0 Hz, 1H), 7.08 (dd, *J* = 9.0, 3.1 Hz, 1H), 7.29 (d, *J* = 3.1 Hz, 1H), 10.37 (s, 1H); ^13^C NMR (126 MHz, CDCl_3_) δ (ppm): 23.6, 28.8, 29.0, 51.5, 52.5, 68.5, 112.1, 113.0, 118.7, 124.7, 151.6, 156.2, 170.5; MS (ESI^+^), *m/z* (%): 280 [M+H]^+^; HRMS (ESI^+^) *calcd.* for C_13_H_18_N_3_O_4_ [M+H]^+^: 280.1292, found 280.1291. Azide (6.79 g, 24.4 mmol, 1.0 equiv) was dissolved in dry THF (234 mL) and TMSOK (10.4 g, 73.2 mmol, 3 equiv; 90% purity) was added. The resulting mixture was stirred at 25°C for 24 h, cooled to 0°C and the pH of the mixture was adjusted to pH = 2 by 10% aq. HCl. The volume of the resulting mixture was in vacuo reduced to ½ of its original volume, and H_2_O (100 mL) was added. The whole mixture was extracted with CH_2_Cl_2_ (2 × 400 mL) and combined organic layers were washed with H_2_O (120 mL), brine (180 mL), dried over MgSO_4_, and organic solvents were removed under reduced pressure. Crude product was dissolved in CH_2_Cl_2_ (20 mL) and hexane (60 mL) was added. Two third of the resulting solvent mixture were removed under reduced pressure and the desired 5-((5-azidopentyl)oxy)-2-hydroxybenzoic acid crystalized off the solution upon prolonged standing (24 h) at 25°C in form of white needles (5.89 g, 91%). Mp = 81-82.5°C; ^1^H NMR (500 MHz, CDCl_3_) δ (ppm): 1.53 – 1.63 (m, 2H), 1.64 – 1.75 (m, 2H), 1.82 (dq, *J* = 8.0, 6.3 Hz, 2H), 3.33 (t, *J* = 6.9 Hz, 2H), 3.95 (t, *J* = 6.3 Hz, 2H), 6.95 (d, *J* = 9.1 Hz, 1H), 7.15 (dd, *J* = 9.1, 3.1 Hz, 1H), 7.35 (d, *J* = 3.1 Hz, 1H), 10.08 (s, 1H); ^13^C NMR (126 MHz, CDCl_3_) δ (ppm): 23.6, 28.9, 29.0, 51.6, 68.6, 110.9, 113.3, 119.0, 126.2, 151.8, 157.0, 173.6; MS (ESI^-^), *m/z* (%): 264 [M-H]^-^; HRMS (ESI^+^) *calcd.* for C_12_H_15_N_3_O_4_Na [M+Na]^+^: 288.0955, found 288.0956. 5-((5-azidopentyl)oxy)-2-hydroxybenzoic acid (0.75 g, 2.83 mmol, 1.0 equiv) was dissolved in EtOAc (14 mL) and 10% of palladium on carbon (3.8 mg, 0.05 equiv) was added. The whole mixture was placed under the hydrogen atmosphere (1.0 atm) and stirred for 24h. The whole mixture was filtered through microfilter (0.5 μm) and the filter was washed with MeOH (2 × 15 mL). Combined filtrates were evaporated under reduced pressure to give 5-((5-aminopentyl)oxy)-2-hydroxybenzoic acid SA-f (0.664 g, 98%) as a viscose oil. ^1^H NMR (500 MHz, DMSO-*d*_*6*_) δ (ppm): 1.42 (p, *J* = 7.7 Hz, 2H), 1.57 (p, *J* = 7.6 Hz, 2H), 1.64 (p, *J* = 7.3, 6.8 Hz, 2H), 2.78 (t, *J* = 7.4 Hz, 2H), 3.80 (t, *J* = 6.2 Hz, 2H), 6.69 (d, *J* = 8.8 Hz, 1H), 6.86 (dd, *J* = 8.8, 3.1 Hz, 1H), 7.36 (d, *J* = 3.2 Hz, 1H); ^13^C NMR (126 MHz, DMSO-*d*_*6*_) δ (ppm): 22.6, 26.8, 28.3, 38.8, 67.7, 114.4, 116.3, 119.5, 120.2, 149.4, 156.4, 171.5; MS (ESI^-^), *m/z* (%): 238 [M-H]^-^; HRMS (ESI^+^) *calcd.* for C_12_H_15_N_3_O_4_Na [M+Na]^+^: 262.1050, found 262.1050. Purity 98+% (LC-MS), R_t_ = 11.93 min.

#### SPR analysis

SPR analysis of SA binding to His-PP2AA1 or His-PP2AA3 was performed with a Biacore T200 instrument (GE Healthcare). A synthesized active SA analog, SA-f, was immobilized on a CM5 sensor chip (GE Healthcare) first: the carboxyl group of the CM5 sensor chip was activated using a mixture of 1-ethyl-3-(3-dimethyl aminopropyl) carbodiimide hydrochloride (EDC) and N-hydroxy-succinimide (NHS) for 7 min at a flow rate of 5 μL/min. After activation, 1 mM of SA-f dissolved in 0.1 M borate buffer (pH 10) was passed over for a period of 3 min at 5 μL/min for immobilization. Then excess reactive groups were inactivated by flowing ethanolamine hydrochloride-NaOH pH 8.5 for 7 min, at 5 μL/min. 1 × PBS buffer (GE Healthcare) was used as running buffer in all assays. To test SA binding of His-PP2AA1 or His-PP2AA3, proteins were diluted in 1 × PBS buffer, and then flowed through the flow cell of sensor chip with SA-f immobilized or through the reference cell. The binding signal was generated by subtracting the signal of reference cell from that generated with the SA-f flow cell. The flow cells were regenerated with flowing 250 mM NaOH solution. Details about the chemical synthesis of SA derivatives are described in the Supplemental Information.

#### Accession Numbers

Sequence data from this article can be found in the *Arabidopsis* Genome Initiative or GenBank/EMBL databases under the following accession numbers: PIN1 (AT1G73590), PIN2 (AT5G57090), NPR1 (AT1G64280), NPR2 (AT4G26120), NPR3 (AT5G45110), NPR4 (AT4G19660), PINOID (AT2G34650), PP2AA1 (AT1G25490), PP2AA2 (AT3G25800), PP2AA3 (AT1G13320), PP2AC3 (AT3G58500), and PP2AC4 (AT2G42500).

### Quantification and Statistical Analysis

For measurement of primary root length and root tip angles, photos were analyzed with ImageJ (https://imagej.nih.gov/ij/download.html). Fluorescence intensity of marker lines were quantified by Fiji (https://fiji.sc/).

Most data plotting and statistics were performed with Graphpad Prism8. A two-tailed t test was used for comparing two datasets. One-way ANOVA with a Tukey multiple comparison test was performed to evaluate the differences of multiple datasets. For root gravitropic responses, polar bar charts were generated by Origin 8.0, and both two-tailed t test and F-test were used to evaluate the mean value and variances respectively.

### Data and Code Availability

This study did not generate/analyze datasets/code.

## Supplemental Information

Document S1. Figures S1–S7 and Table S1

Document S2. Article plus Supplemental Information
