# Supplementary material for: Salicylic Acid Targets Protein Phosphatase 2A to Attenuate Growth in Plants
Source: Curr Biol. 2020 Feb 3;30(3):381–395.e8. doi: 10.1016/j.cub.2019.11.058 (PMC6997888; doi:10.1016/j.cub.2019.11.058)
Supplement: Document S1. Figures S1–S7 and Table S1 [file mmc1.pdf]

Current Biology, Volume *30*

Supplemental Information

Salicylic Acid Targets Protein Phosphatase 2A to Attenuate Growth in Plants

Shutang Tan, Melinda Abas, Inge Verstraeten, Matouš Glanc, Gergely Molnár, Jakub Hajný, Pavel Lasák, Ivan Petřík, Eugenia Russinova, Jan Petrášek, Ondřej Novák, Jiří Pospíšil, and Jiří Friml

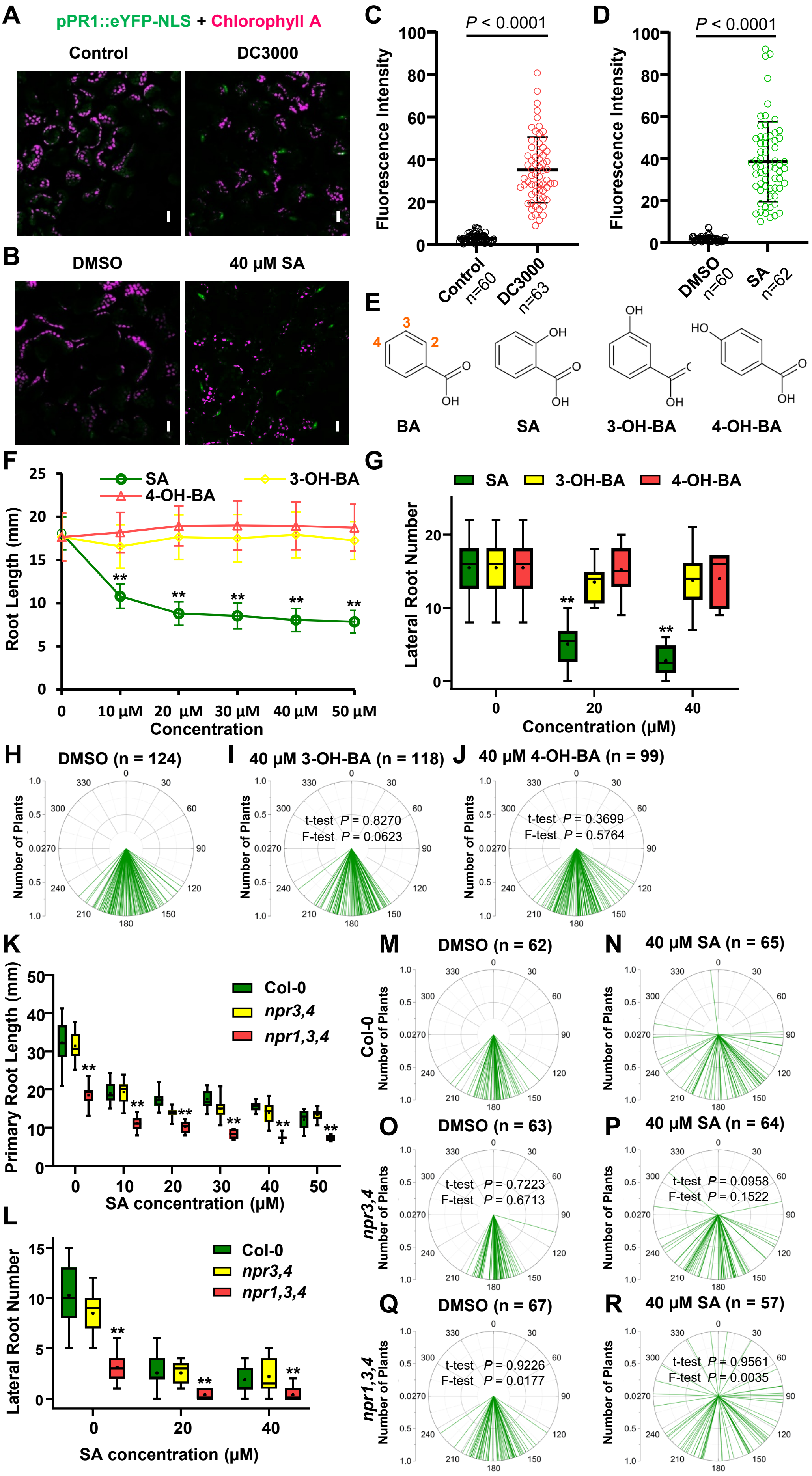
A
pPR1::eYFP-NLS + Chlorophyll A
Control
DC3000
B
DMSO
40 μM SA
C
P < 0.0001
Fluorescence Intensity
Control n=60
DC3000 n=63
D
P < 0.0001
Fluorescence Intensity
DMSO n=60
SA n=62
E
BA
SA
3-OH-BA
4-OH-BA
F
SA
3-OH-BA
4-OH-BA
Root Length (mm)
10 μM 20 μM 30 μM 40 μM 50 μM
Concentration
G
SA
3-OH-BA
4-OH-BA
Lateral Root Number
Concentration (μM)
H
DMSO (n = 124)
I
40 μM 3-OH-BA (n = 118)
t-test P = 0.8270
F-test P = 0.0623
J
40 μM 4-OH-BA (n = 99)
t-test P = 0.3699
F-test P = 0.5764
Number of Plants
K
Primary Root Length (mm)
Col-0
npr3,4
npr1,3,4
SA concentration (μM)
L
Lateral Root Number
Col-0
npr3,4
npr1,3,4
SA concentration (μM)
M
DMSO (n = 62)
N
40 μM SA (n = 65)
Col-0
O
DMSO (n = 63)
P
40 μM SA (n = 64)
npr3,4
t-test P = 0.7223
F-test P = 0.6713
t-test P = 0.0958
F-test P = 0.1522
Q
DMSO (n = 67)
R
40 μM SA (n = 57)
npr1,3,4
t-test P = 0.9226
F-test P = 0.0177
t-test P = 0.9561
F-test P = 0.0035

Figure S1. Structural isomers and the specific role of SA in the root growth regulation. Related to Figures 1 & 2.

(A-D) Induced *pPR1::eYFP-NLS* expression by *P. syringae* DC3000 (A, C) or SA (B, D) in cotyledons. (A, C) 5-d-old *pPR1::eYFP-NLS* seedlings were treated with *P. syringae* DC3000 (OD_{600} = 0.01, ~5×10^6 CFU/mL) or with resuspension buffer (control) for 48 h, and were then imaged by CLSM. Scale bars, 10 µm. (B, D) Induced *pPR1::eYFP-NLS* expression by SA in cotyledons. 5-d-old *pPR1::eYFP-NLS* seedlings were transferred to plates with DMSO or 40 µM SA for 24h, and were then imaged by CLSM. Scale bars, 10 µm. For quantification, the average GFP fluorescence of 5-10 representative cells from 10 seedlings for each treatment was measured by Fiji. The data points were shown as dot plots. Dots represent individual values, and lines indicate mean ± SD. *P* values were calculated by a two-tailed t-test.

(E) Structures of benzoic acid analogues, including benzoic acid (BA), 2-hydroxybenzoic acid (also known as ortho-salicylic acid, SA), 3-hydroxybenzoic acid (3-OH-BA, also known as meta-salicylic acid), and 4-hydroxybenzoic acid (4-OH-BA, also known as para-salicylic acid). Chemical structures were illustrated with the ChemSketch program.

(F) 3-OH-BA and 4-OH-BA do not inhibit primary root elongation. Root length of 7-d-old Col-0 seedlings grown on MS plates containing different concentrations of SA, 3-OH-BA or 4-OH-BA was measured. Note that the same DMSO control was used for all the indicated chemicals. n = 100-129. **, $P < 0.01$, by a two-tailed t-test.

(G) 3-OH-BA and 4-OH-BA do not repress lateral root formation. The lateral root number of 10-d-old plants was counted. The same DMSO control was used for all the indicated chemicals. n = 10-22. **, $P < 0.01$, by a two-tailed t-test.

(H-J) 3-OH-BA and 4-OH-BA do not have a significant effect on root gravitropism. The root tip angles of 7-d-old Col-0 seedlings on different plates were measured. *P* values were calculated by a two-tailed t-test to evaluate the mean value and by a further F-test to indicate differences of variances.

(K) SA inhibited primary root elongation, which was not suppressed by NPR deficiency. Root length of 7-d-old Col-0, *npr3,4* and *npr1,3,4* seedlings grown on MS plates containing different concentrations of SA was measured. n = 10-26. **, $P < 0.01$. *P* values were calculated by one-way ANOVA with a Tukey multiple comparison test, compared to Col-0 in each treatment.

(L) Inhibition of lateral root formation by SA does not depend on *NPRs*. The lateral root number of 10-d-old Col-0, *npr3,4* and *npr1,3,4* plants was counted. n = 9-24. **, $P < 0.01$. *P* values were calculated by one-way ANOVA with a Tukey multiple comparison test, compared to Col-0.

(M-R) SA repressed root gravitropism independently of *NPRs*. The angles of root tips of 7-d-old Col-0 (M, N), *npr3,4* (O, P) and *npr1,3,4* (Q, R) seedlings were measured. *P* values were calculated by a two-tailed t-test to evaluate the mean value and by a further F-test to indicate differences of variances.

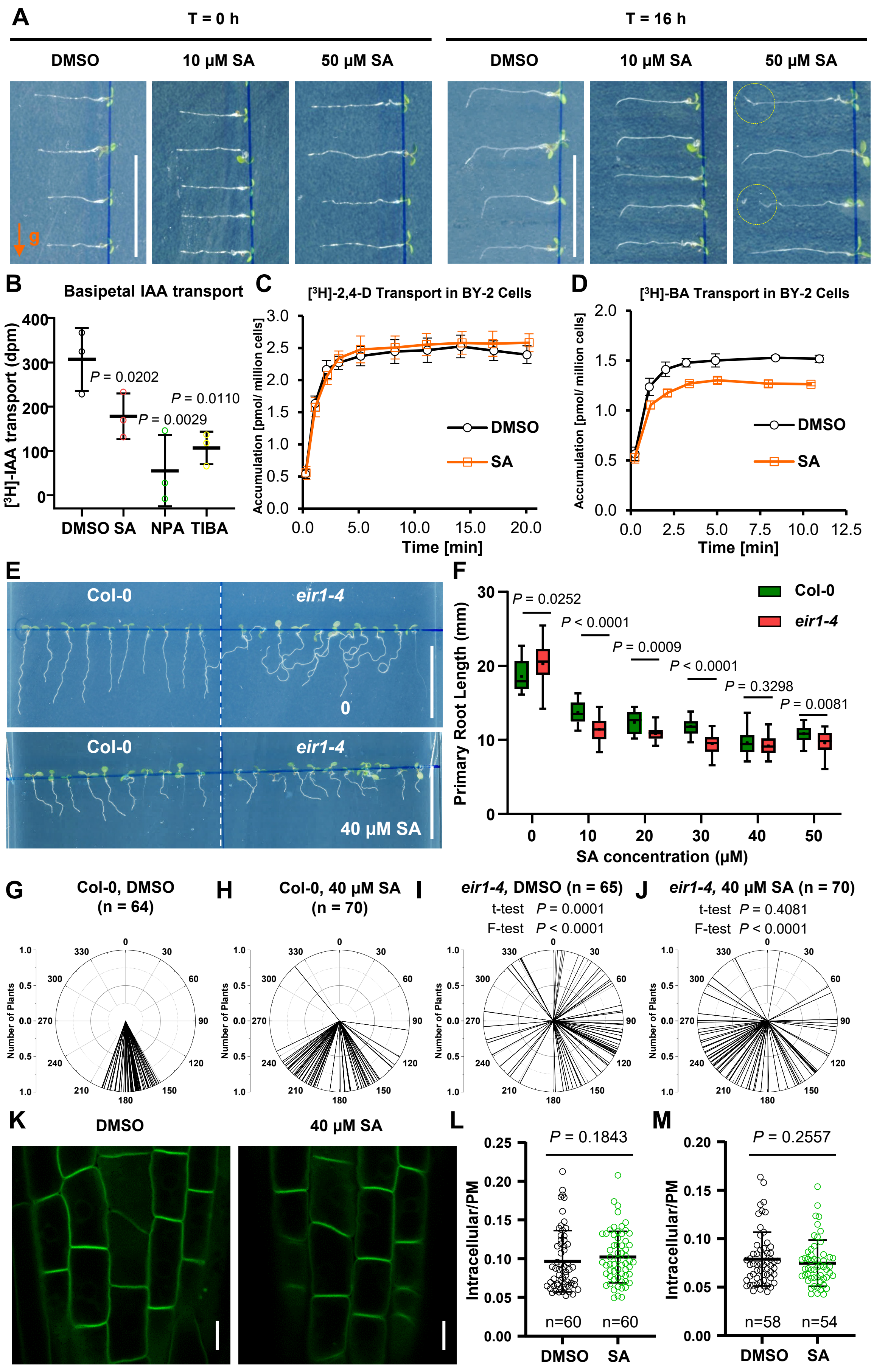
A
T = 0 h
T = 16 h
DMSO
10 μM SA
50 μM SA
DMSO
10 μM SA
50 μM SA
g
B
Basipetal IAA transport
[³H]-IAA transport (dpm)
400
300
200
100
0
P = 0.0202
P = 0.0110
P = 0.0029
DMSO SA NPA TIBA
C
[³H]-2,4-D Transport in BY-2 Cells
Accumulation [pmol/ million cells]
3.0
2.5
2.0
1.5
1.0
0.5
0.0
DMSO
SA
0.0 5.0 10.0 15.0 20.0
Time [min]
D
[³H]-BA Transport in BY-2 Cells
Accumulation [pmol/ million cells]
2.0
1.5
1.0
0.5
0.0
DMSO
SA
0.0 2.5 5.0 7.5 10.0 12.5
Time [min]
E
Col-0
eir1-4
0
Col-0
eir1-4
40 μM SA
F
Primary Root Length (mm)
30
20
10
0
P = 0.0252
P < 0.0001
P = 0.0009
P < 0.0001
P = 0.3298
P = 0.0081
Col-0
eir1-4
0 10 20 30 40 50
SA concentration (μM)
G
Col-0, DMSO
(n = 64)
H
Col-0, 40 μM SA
(n = 70)
I
eir1-4, DMSO (n = 65)
t-test P = 0.0001
F-test P < 0.0001
J
eir1-4, 40 μM SA (n = 70)
t-test P = 0.4081
F-test P < 0.0001
Number of Plants
1.0
0.5
0.0
0.5
1.0
0
30
60
90
120
150
180
210
240
270
300
330
K
DMSO
40 μM SA
L
P = 0.1843
Intracellular/PM
0.25
0.20
0.15
0.10
0.05
0.00
n=60 n=60
DMSO SA
M
P = 0.2557
Intracellular/PM
0.20
0.15
0.10
0.05
0.00
n=58 n=54
DMSO SA

Figure S2. Specific SA action on root gravitropic response and auxin export, in a PIN-dependent manner. Related to Figure 3.

(A) Representative images showing the gravitropic response of WT seedlings on different concentrations of SA. 5-d-old seedlings were transferred to indicated plates, and grown for additional 16 h. DMSO as the solvent control. Agravitropic roots are marked. Scale bars, 2 cm.

(B) SA inhibited the basipetal transport of [^{3}H]-IAA in etiolated hypocotyls. DMSO, 10 µM NPA, 10 µM TIBA, and 500 µM SA were added to both the [^{3}H]-IAA droplets and the medium. 15 seedlings were pooled as a biological replicate; n = 3. Dots represent individual values, and lines indicate mean ± SD. *P* values were calculated by one-way ANOVA with a Tukey multiple comparison test, compared to DMSO.

(C-D) SA has no effect on the accumulation of [^{3}H]-2,4-D (C) or [^{3}H]-BA (D) in tobacco BY-2 cells, suggesting no effect on export, as controls for [^{3}H]-IAA. DMSO (the solvent control) and 200 µM SA were added to the cell culture, and then the radioactivity of ^{3}H was measured at indicated timepoints after the addition of [^{3}H]-2,4-D (C) or [^{3}H]-BA (D) to the DMSO- and SA-treated cell cultures. n = 3.

(E) Representative images show the sensitivity of *eir1-4* to SA. Col-0 and *eir1-4* seedlings were grown on plates with different concentrations of SA for 7 d. Scale bars, 2 cm.

(F) *eir1-4* shows slightly increased sensitivity to SA in root growth inhibition. Col-0 and *eir1-4* seedlings were grown on plates with concentrations of SA for 7 d, and the primary root length was measured. n = 16-23; *P* values were calculated by a two-tailed t-test for indicated pairs of Col-0 and *eir1-4* at a certain concentration of SA.

(G-J) *eir1-4* showed agravitropic roots, which were not further enhanced by SA treatment. Col-0 and *eir1-4* seedlings were grown on plates with different concentrations of SA for 7 d, and the root tip angles were measured by Image J, and shown by polar bar charts. *P* values were calculated by a two-tailed t-test to evaluate the mean value and by a further F-test to indicate differences of variances. The *eir1-4* groups were compared with Col-0 under treatment with the same concentration of SA respectively.

(K) The localization of AUX1-YFP. Plants were grown for 4 d on DMSO or 40 µM SA. Scale bars, 10 µm.

(L-M) Quantification of AUX1-YFP subcellular distribution revealed by its intracellular/PM fluorescence ratio. The average AUX1-YFP fluorescence of the intracellular area and PM of 5-10 representative cells from 10 seedlings for each treatment was measured by Fiji. The data points were shown as dot-plots. Dots represent individual values, and lines indicate mean ± s.d.. *P* values were calculated by a two-tailed t-test. (L) 4-d-old seedling grown on plates with 40 µM SA are shown in (K); (M) 4-d-old seedling were treated with 40 µM SA for 12 h.

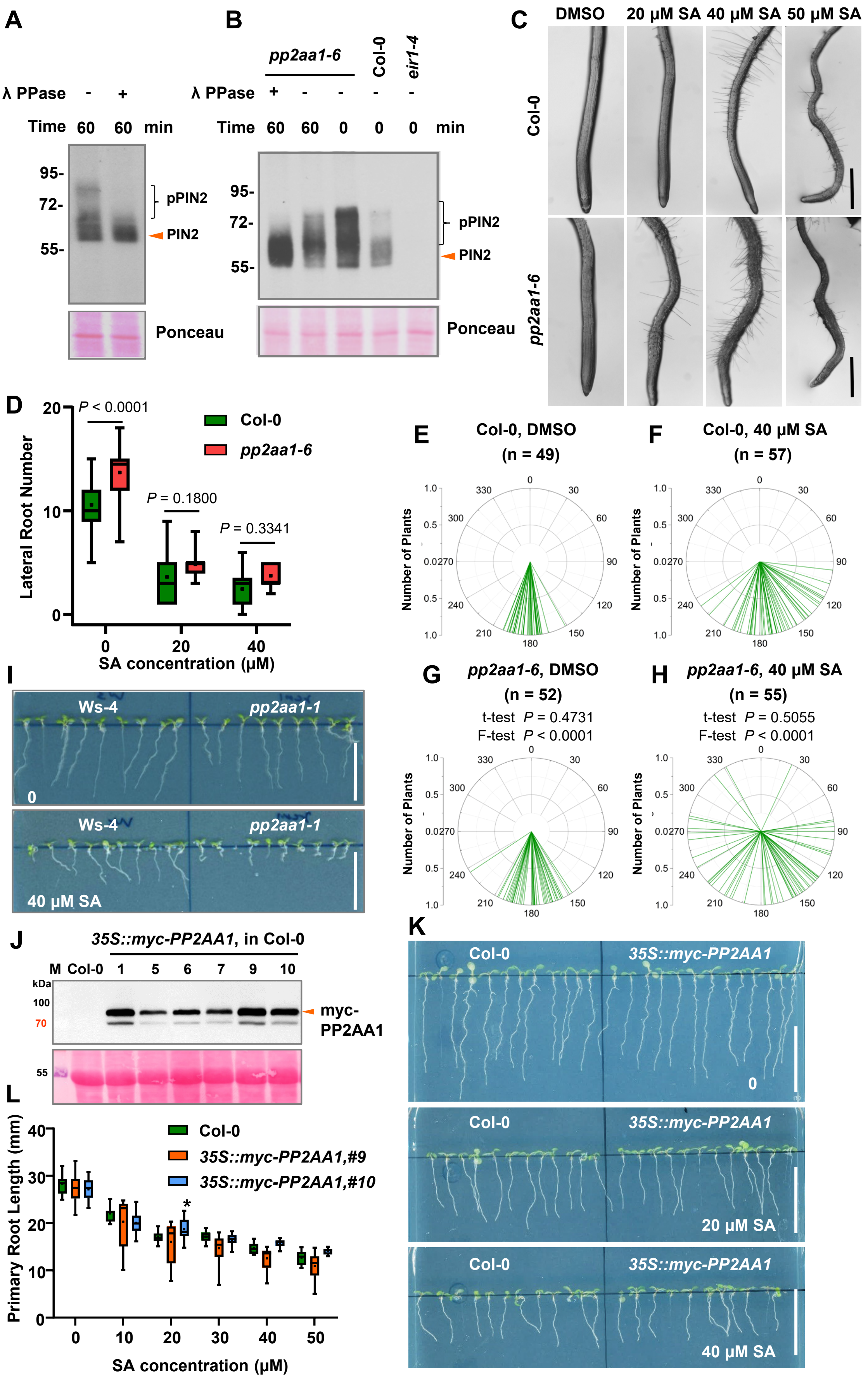
A
λ PPase - +
Time 60 60 min
95-
72-
55-
pPIN2
PIN2
Ponceau
B
pp2aa1-6
Col-0
eir1-4
λ PPase + - - - -
Time 60 60 0 0 0 min
95-
72-
55-
pPIN2
PIN2
Ponceau
C
DMSO 20 μM SA 40 μM SA 50 μM SA
Col-0
pp2aa1-6
D
P < 0.0001
P = 0.1800
P = 0.3341
Col-0
pp2aa1-6
Lateral Root Number
SA concentration (μM)
E
Col-0, DMSO
(n = 49)
F
Col-0, 40 μM SA
(n = 57)
G
pp2aa1-6, DMSO
(n = 52)
t-test P = 0.4731
F-test P < 0.0001
H
pp2aa1-6, 40 μM SA
(n = 55)
t-test P = 0.5055
F-test P < 0.0001
Number of Plants
I
Ws-4
pp2aa1-1
0
40 μM SA
J
35S::myc-PP2AA1, in Col-0
M Col-0 1 5 6 7 9 10
kDa
100
70
55
myc-PP2AA1
K
Col-0
35S::myc-PP2AA1
0
20 μM SA
40 μM SA
L
Col-0
35S::myc-PP2AA1,#9
35S::myc-PP2AA1,#10
Primary Root Length (mm)
SA concentration (μM)

Figure S3. Deficiency of the PP2A A subunit, PP2AA1 (RCN1), leads to SA hypersensitivity, and SA treatment inhibits PP2A activity *in planta*. Related to Figure 4.

(A) PIN2 phosphorylation gave rise to shifted smears, revealed by dephosphorylation of PIN2 by lambda phosphatase (λ PPase) *in vitro*. Total membrane extracts were incubated with or without 2 U of λ PPase for the indicated time, and samples were then analysed by Western blot with an anti-PIN2 antibody. Upper, anti-PIN2; bottom, Ponceau staining.

(B) Increase of PIN2 phosphorylation in *pp2aa1-6*. Total membrane extracts from Col-0, *pp2aa1-6*, and *eir1-4* were incubated with or without 5 U λ PPase for the indicated time, and samples were analysed by Western blot with an anti-PIN2 antibody. Upper, anti-PIN2; bottom, Ponceau staining.

(C) Close views of the morphology of Col-0 and *pp2aa1-6* roots under different concentrations of SA. Seedlings were observed by a differential interference contrast (DIC) microscopy. Scale bars, 1 mm.

(D) Sensitivity of *pp2aa1-6* to SA in terms of inhibiting lateral root formation. Col-0 and *pp2aa1-6* seedlings were grown on plates containing different concentrations of SA for 10 d, and the number of emerged lateral root was counted. *P* values were calculated by a two-tailed t-test.

(E-H) Following Figure 4F. *pp2aa1-6* is hypersensitive to SA in terms of interfering with the gravitropic response. Col-0 (E-F) and *pp2aa1-6* (G-H) seedlings were grown on plates containing different concentrations of SA for 7 d, and the root tip angles were measured by Image J, and shown as polar bar charts. *P* values were calculated by a two-tailed t-test to evaluate the mean value and by a further F-test to indicate differences of variances. The *pp2aa1-6* groups were compared with Col-0 under treatment with the same concentration of SA respectively.

(I) Representative images showing hypersensitivity of *pp2aa1-1* to SA. WT (Ws-4) and *pp2aa1-1* seedlings were grown on plates with different concentrations of SA for 7 d. Scale bars, 2 cm.

(J) Western blot verified the expression of myc-PP2AA1. 7-d-old seedlings were subjected to protein extraction and the subsequent Western blot with an anti-myc antibody (1:2000). Upper panel, anti-myc; lower panel, Ponceau staining to show the loading. Lines 9 and 10 (T_3 generation, homozygous lines) were used for further analysis.

(K) Representative images showing the sensitivity of *35S::myc-PP2AA1* to SA. Col-0 and *35S::myc-PP2AA1* seedlings were grown on plates with different concentrations of SA for 7 d. Scale bars, 2 cm.

(L) *35S::myc-PP2AA1* did not show any difference in sensitivity to SA in root growth inhibition. Col-0 and *35S::myc-PP2AA1* seedlings were grown on plates with concentrations of SA for 7 d, and the primary root length was measured. n = 11-30; *, $P <$ 0.05, by one-way ANOVA with a Tukey multiple comparison test, compared to Col-0.

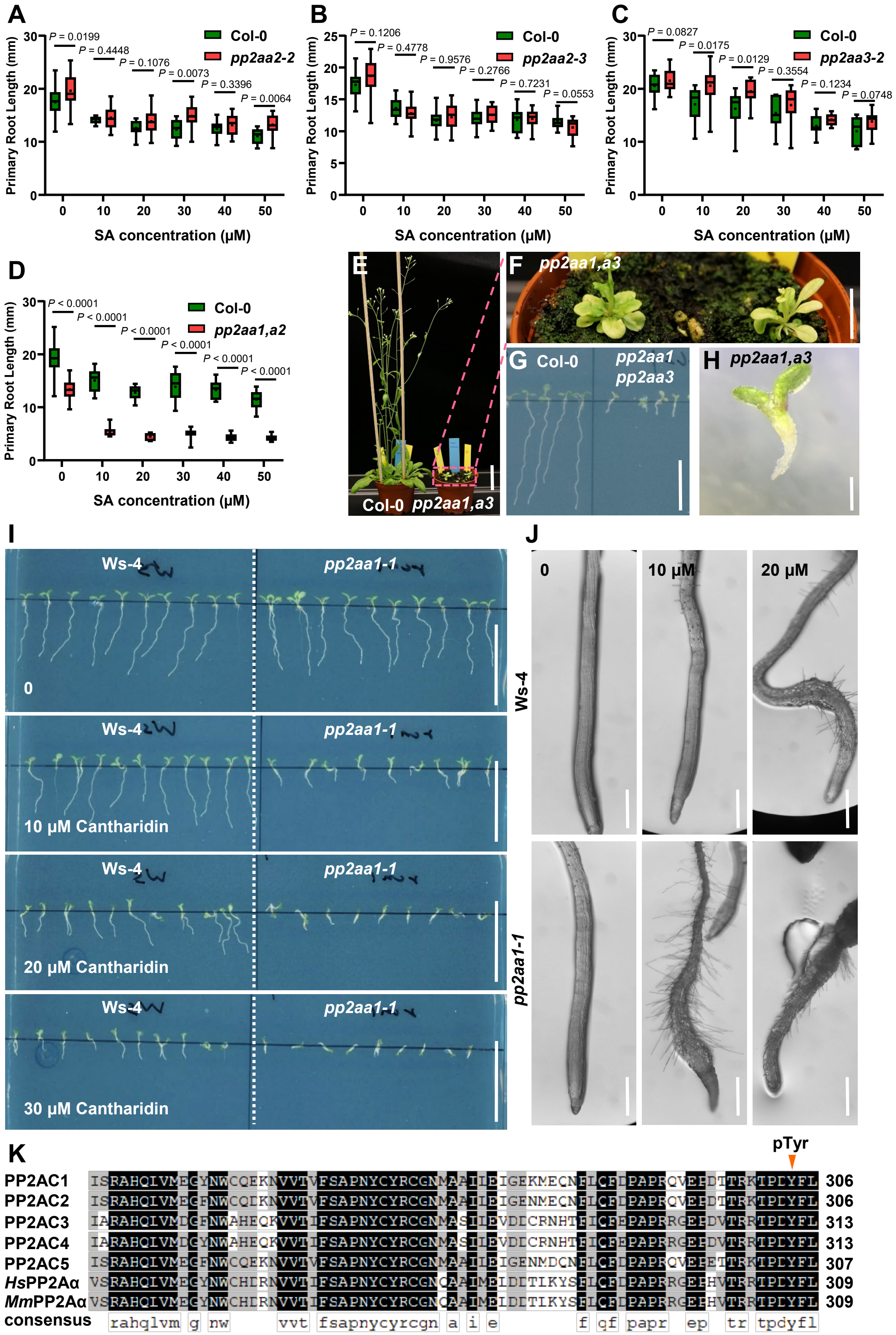
A
Col-0
pp2aa2-2
Primary Root Length (mm)
SA concentration (μM)
P = 0.0199
P = 0.4448
P = 0.1076
P = 0.0073
P = 0.3396
P = 0.0064
B
Col-0
pp2aa2-3
P = 0.1206
P = 0.4778
P = 0.9576
P = 0.2766
P = 0.7231
P = 0.0553
C
Col-0
pp2aa3-2
P = 0.0827
P = 0.0175
P = 0.0129
P = 0.3554
P = 0.1234
P = 0.0748
D
Col-0
pp2aa1,a2
P < 0.0001
E
Col-0 pp2aa1,a3
F pp2aa1,a3
G Col-0
pp2aa1
pp2aa3
H pp2aa1,a3
I
Ws-4
pp2aa1-1
0
10 μM Cantharidin
20 μM Cantharidin
30 μM Cantharidin
J
0
10 μM
20 μM
Ws-4
pp2aa1-1
K
pTyr
PP2AC1 306
PP2AC2 306
PP2AC3 313
PP2AC4 313
PP2AC5 307
HsPP2Aα 309
MmPP2Aα 309
consensus
rahqlvm g nw vvt fsapnycyrcgn a i e f qf papr ep tr tpdyfl

Figure S4. SA sensitivity of the loss-of-function mutants of PP2A subunits, and deficiency of the PP2A A subunit, PP2AA1 (RCN1), leads to hypersensitivity to a known PP2A inhibitor, cantharidin. Related to Figures 4 & 5.

(A-D) The sensitivity of different PP2A mutants to SA in primary root elongation. Col-0, *pp2aa2-2*, *pp2aa2-3* (a knock-down line), *pp2aa3-2*, and *pp2aa1 pp2aa2-3* (short as *pp2aa1,a2*) seedlings were grown on plates with different concentrations of SA for 7 d, and the primary root length was measured. (A) n = 11-27，(B) n = 12-30，(C) n = 10-28，(D) n = 13-28. *P* values were calculated by a two-tailed t-test for indicated pairs of Col-0 and *pp2aa1, a3* at the given concentration of SA.

(E-H) The double mutant of *pp2aa1 pp2aa3* exhibited deficiency in growth and development with severe root defects, reminiscent of SA- or cantharidin- treatment. Scale bar, 5 cm (E), 1 cm (F), 1 cm (G), and 1 mm (H) respectively.

(I) Representative images showing the hypersensitivity of *pp2aa1-1* to cantharidin. Ws-4 and *pp2aa1-1* seedlings were grown on plates containing different concentrations of cantharidin for 7 d. Scale bars, 2 cm.

(J) Close view of the morphology of Ws-4 and *pp2aa1-1* roots under different concentrations of cantharidin. Scale bars, 1 mm.

(K) Sequence alignment of *Arabidopsis* PP2A C subunits, with their homologues in human (*Homo sapiens*) and mice (*Mus musculus*). All the sequences share 87.13% similarity. The arrowhead indicates the conserved phosphorylation site, which is responsible for PP2Ac activity and is recognized by the pY307-PP2Ac antibody.

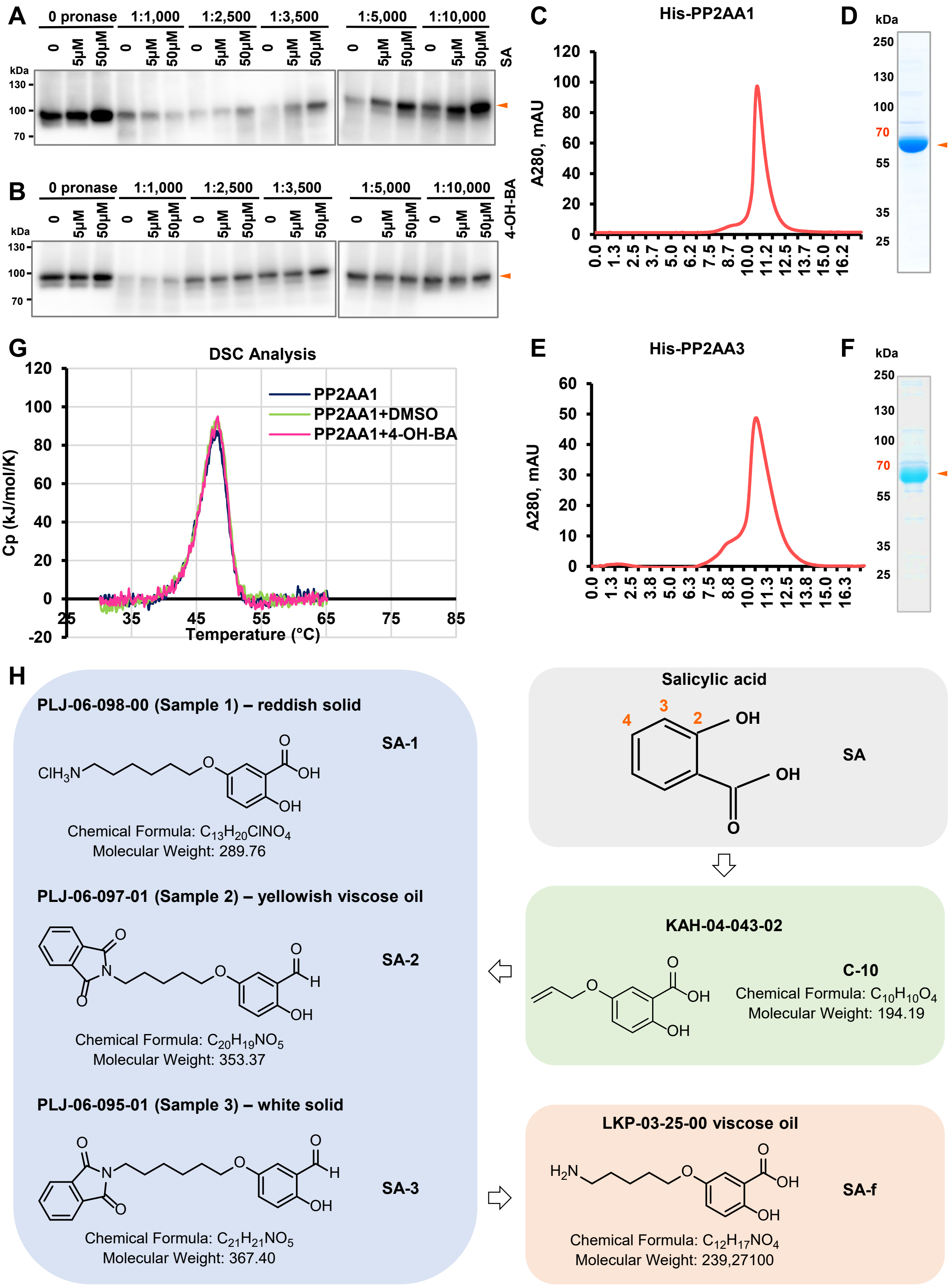
A
0 pronase
1:1,000
1:2,500
1:3,500
1:5,000
1:10,000
0
5µM
50µM
SA
kDa
130
100
70
B
4-OH-BA
C
His-PP2AA1
A280, mAU
120
100
80
60
40
20
0
0.0
1.3
2.5
3.7
5.0
6.2
7.5
8.7
10.0
11.2
12.5
13.7
15.0
16.2
D
kDa
250
130
100
70
55
35
25
E
His-PP2AA3
60
50
40
30
20
10
0.0
1.3
2.5
3.8
5.0
6.3
7.5
8.8
10.0
11.3
12.5
13.8
15.0
16.3
F
G
DSC Analysis
Cp (kJ/mol/K)
PP2AA1
PP2AA1+DMSO
PP2AA1+4-OH-BA
-20
25
35
45
55
65
75
85
Temperature (°C)
H
PLJ-06-098-00 (Sample 1) – reddish solid
SA-1
ClH3N
O
OH
Chemical Formula: C13H20ClNO4
Molecular Weight: 289.76
PLJ-06-097-01 (Sample 2) – yellowish viscose oil
SA-2
N
H
Chemical Formula: C20H19NO5
Molecular Weight: 353.37
PLJ-06-095-01 (Sample 3) – white solid
SA-3
Chemical Formula: C21H21NO5
Molecular Weight: 367.40
Salicylic acid
SA
2
3
4
KAH-04-043-02
C-10
Chemical Formula: C10H10O4
Molecular Weight: 194.19
LKP-03-25-00 viscose oil
SA-f
H2N
Chemical Formula: C12H17NO4
Molecular Weight: 239,27100

Figure S5. DARTS assay suggests potential binding of SA to PP2AA1, protein purification of His-PP2AA1 and His-PP2AA3 by Size exclusion chromatography (SEC), and the design flow of the SA analogue, SA-f, for the SPR. Related to Figure 6.

(A-B) DARTS assay suggests PP2AA1 is potential target of SA. *pPP2AA1::PP2AA1-GFP* seedlings were used for the protein isolation, and Samples were treated with DMSO (mock) and SA (0, 5 μM, and 50 μM respectively, in A), with 4-OH-BA as a negative control (B), and digested by different concentrations of pronase.

(C) SEC purification of His-PP2AA1 with a Superdex 200 increase column. A representative run is shown here to indicate the purity of recombinant His-PP2AA1 used for DSC and SPR analyses.

(D) SDS-PAGE to check the quality of His-PP2AA1, visualized by CBB staining.

(E) SEC purification of His-PP2AA3.

(F) SDS-PAGE to check the quality of His-PP2AA3, by CBB staining.

(G) DSC analysis of the effect of 4-OH-BA, an inactive SA isomer, on His-PP2AA1 stability. 5 μM of purified His-PP2AA1 protein were added with or without DMSO, or 50 μM 4-OH-BA, and were then analysed by DSC. Tm = 47.57°C, 47.69°C, and 47.69°C, for His-PP2AA1, His-PP2AA1+DMSO, and His-PP2AA1+4-OH-BA respectively.

(H) Workflow for the design of the synthetic SA analogue, SA-f, which can be immobilized on a CM-5 SPR sensor chip. Multiple SA analogues with different groups at different positions of the benzoic ring were synthesized, tested, with C-10 coming out as the best one with activity and the possibility to immobilize it. Further analysis for SA-1/2/3 with a linker and a group to mimic the surface of the matrix of SPR sensor chips, revealed that the linker does not affect the bioactivity of SA.

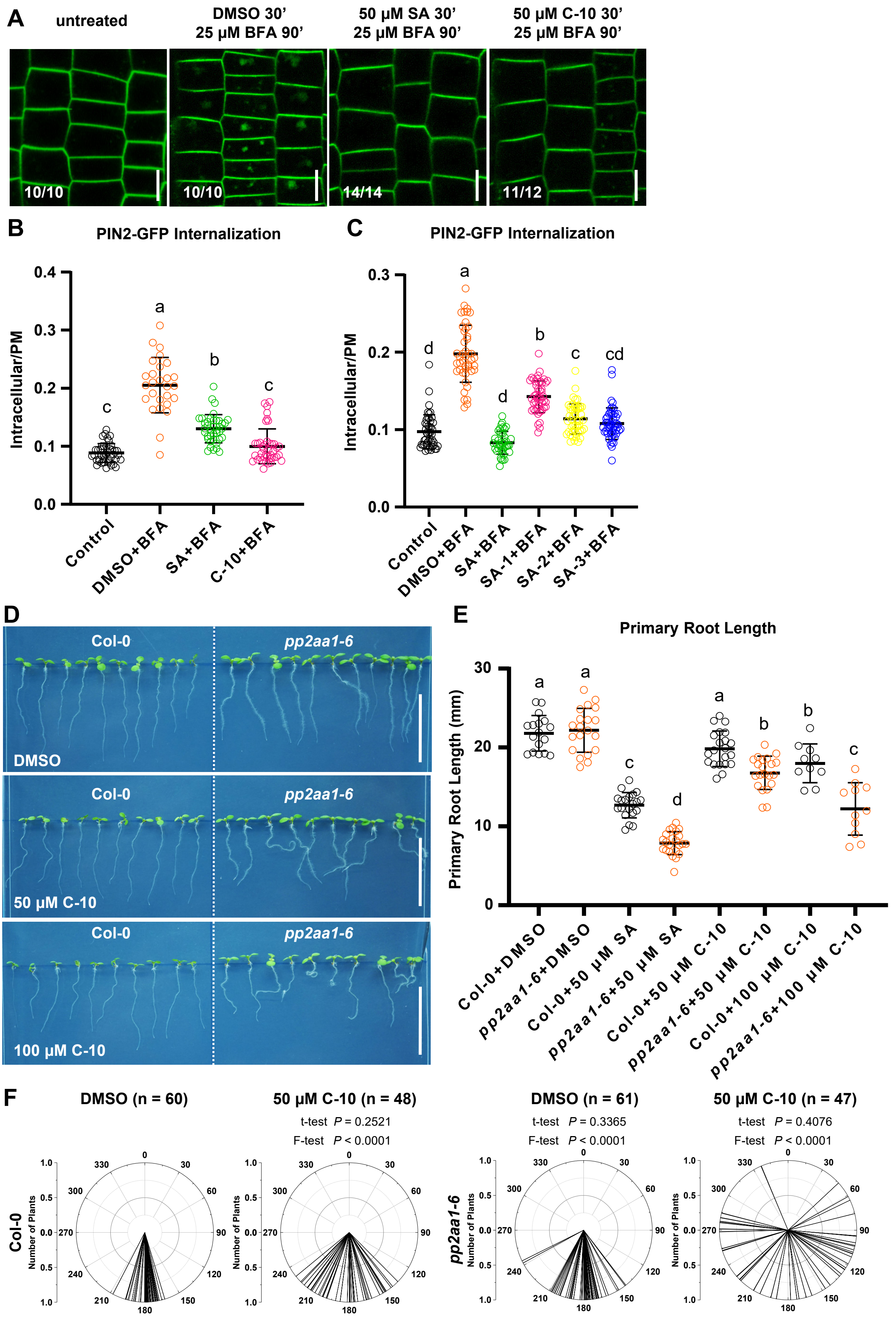

A
untreated
DMSO 30'
25 µM BFA 90'
50 µM SA 30'
25 µM BFA 90'
50 µM C-10 30'
25 µM BFA 90'
10/10
10/10
14/14
11/12
B
PIN2-GFP Internalization
Intracellular/PM
Control
DMSO+BFA
SA+BFA
C-10+BFA
C
PIN2-GFP Internalization
Intracellular/PM
Control
DMSO+BFA
SA+BFA
SA-1+BFA
SA-2+BFA
SA-3+BFA
D
Col-0
pp2aa1-6
DMSO
50 µM C-10
100 µM C-10
E
Primary Root Length
Primary Root Length (mm)
Col-0+DMSO
pp2aa1-6+DMSO
Col-0+50 µM SA
pp2aa1-6+50 µM SA
Col-0+50 µM C-10
pp2aa1-6+50 µM C-10
Col-0+100 µM C-10
pp2aa1-6+100 µM C-10
F
DMSO (n = 60)
50 µM C-10 (n = 48)
t-test P = 0.2521
F-test P < 0.0001
DMSO (n = 61)
t-test P = 0.3365
F-test P < 0.0001
50 µM C-10 (n = 47)
t-test P = 0.4076
F-test P < 0.0001
Col-0
pp2aa1-6
Number of Plants

Figure S6. Bioactivity test of the synthetic SA analogues. Related to Figure 6.

(A-B) Cellular activity of the synthetic compound, C-10, with the SA moiety, in terms of inhibiting BFA (brefeldin A) body formation. 4-d-old *pPIN2::PIN2-GFP* seedlings were treated with indicated chemicals and imaged by CLSM. (A), representative images, Scale bars, 10 μm; (B), quantification of the BFA body formation by calculating the intracellular/PM ratio for the PIN2-GFP fluorescence intensity.

(C) Cellular activity of the synthetic compounds, SA-1 to SA-3, in terms of inhibiting BFA body formation. 4-d-old *pPIN2::PIN2-GFP* seedlings were treated with indicated chemicals and imaged by CLSM. Quantification of the BFA body formation by calculating the intracellular/PM ratio for the PIN2-GFP fluorescence intensity.

Dots represent individual values, and lines indicate mean ± SD. (B) n = 27-42; (C) n = 41-50. Different letters represent significant difference, $P < 0.05$, by one-way ANOVA with a Tukey multiple comparison test.

(D) Physiological activity of the synthetic SA analogue C-10, in terms of root morphology. 7-d-old Col-0 seedlings were grown on plates with indicated chemicals. Scale bars, 2 cm.

(E) C-10 inhibits primary root elongation. Dots represent individual values, and lines indicate mean ± SD. n =10-23; *P* values were calculated by a two-tailed t-test.

(F) Treatment with C-10 gave rise to less gravitropic roots. The angles of root tips were measured by Image J, and shown as polar bar charts. *P* values were calculated by a two-tailed t-test to evaluate the mean value and by a further F-test to indicate differences of variances. For Col-0, C-10 treatments were compared with the DMSO control, and the *pp2aa1-6* groups were compared with Col-0 under treatment with the same concentration of C-10 respectively.

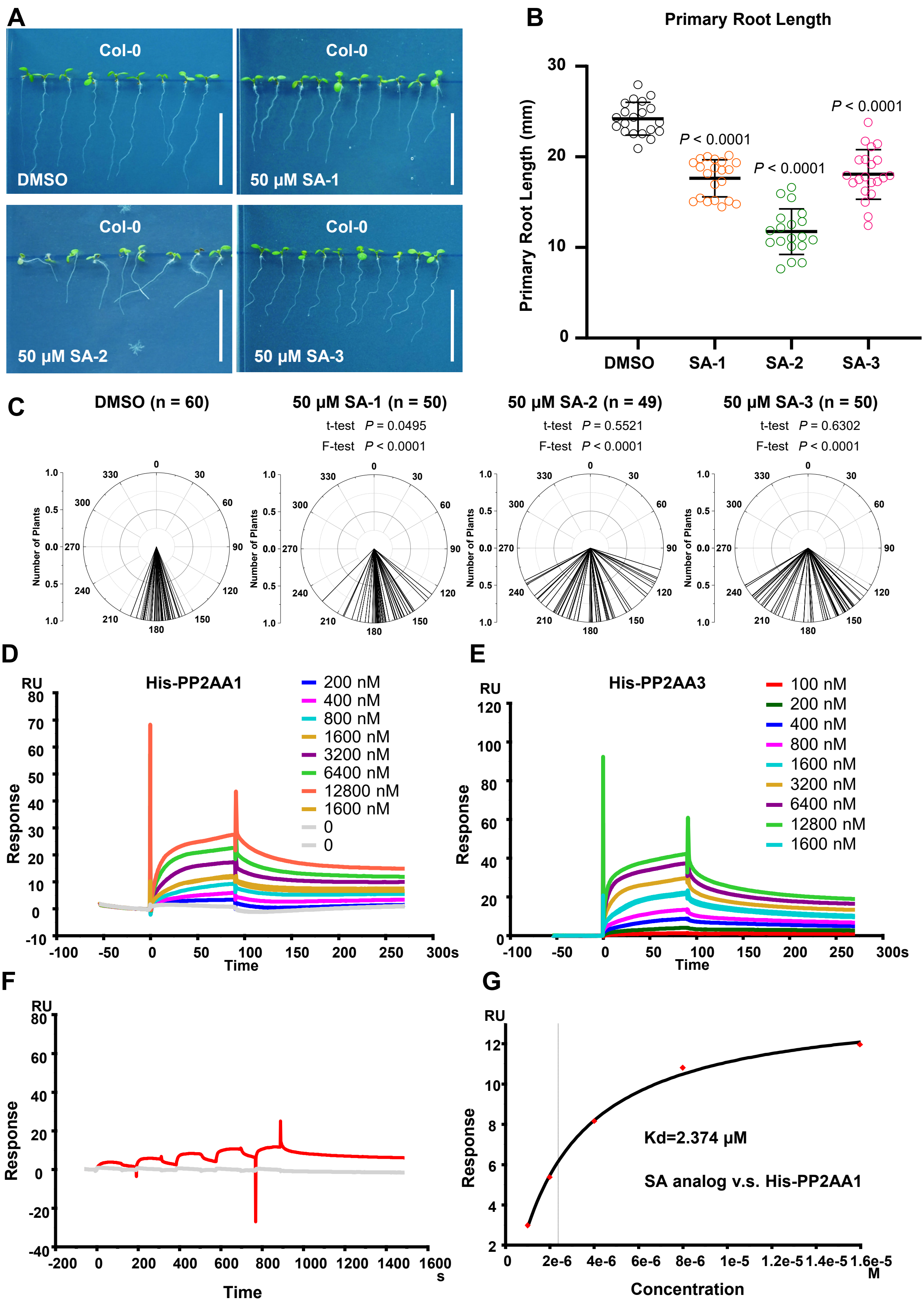
A
Col-0
DMSO
Col-0
50 µM SA-1
Col-0
50 µM SA-2
Col-0
50 µM SA-3
B
Primary Root Length
Primary Root Length (mm)
30
20
10
0
P < 0.0001
P < 0.0001
P < 0.0001
DMSO
SA-1
SA-2
SA-3
C
DMSO (n = 60)
50 µM SA-1 (n = 50)
t-test P = 0.0495
F-test P < 0.0001
50 µM SA-2 (n = 49)
t-test P = 0.5521
F-test P < 0.0001
50 µM SA-3 (n = 50)
t-test P = 0.6302
F-test P < 0.0001
Number of Plants
0
30
60
90
120
150
180
210
240
270
300
330
D
His-PP2AA1
RU
Response
Time
200 nM
400 nM
800 nM
1600 nM
3200 nM
6400 nM
12800 nM
1600 nM
0
0
-100 -50 0 50 100 150 200 250 300s
E
His-PP2AA3
RU
Response
Time
100 nM
200 nM
400 nM
800 nM
1600 nM
3200 nM
6400 nM
12800 nM
1600 nM
-100 -50 0 50 100 150 200 250 300s
F
RU
Response
Time
-200 0 200 400 600 800 1000 1200 1400 1600 s
G
RU
Response
Concentration
Kd=2.374 µM
SA analog v.s. His-PP2AA1
0 2e-6 4e-6 6e-6 8e-6 1e-5 1.2e-5 1.4e-5 1.6e-5 M

Figure S7. Physiological test of the SA analogues, and additional data for the binding between SA and His-PP2AA1. Related to Figure 6.

(A) Physiological activity of the synthetic SA analogues SA-1 to SA-3, in terms of root morphology. 7-d-old Col-0 seedlings were grown on plates with indicated chemicals. Scale bars, 2 cm.

(B) SA analogues (SA-1 to SA-3) inhibit the primary root elongation. Dots represent individual values, and lines indicate mean ± SD. n =17-21; *P* values were calculated by a two-tailed t-test.

(C) Treatment with SA analogues (SA-1 to SA-3) gave rise to less gravitropic roots. The angles of root tips were measured by Image J, and shown as polar bar charts. *P* values were calculated by a two-tailed t-test to evaluate the mean value and by a further F-test to indicate differences of variances in comparison to mock treatment.

(D) SPR assay reveals the binding of His-PP2AA1 to SA. The sensorgram shows the kinetics for the interaction between His-PP2AA1 and SA, used for generating Figure 6C.

(E) SPR assay reveals the binding of His-PP2AA3 to SA. Sensorgram showing the kinetics for the interaction between His-PP2AA3 and SA.

(F-G) SPR revealing the binding between SA and His-PP2AA1. Single cycle binding kinetics was conducted, without regeneration between different concentrations of His-PP2AA1. 0.1% BSA (~15 μM) was included in the His-PP2AA1 flow. (F), sensorgram; (G), plotted by the values at steady state. A Kd value of 2.374 μM was detected.

**Primers**	**Oligonucleotide (5’ to 3’)**	**use**
**For Genotyping**		
**Oligo Name**	**Sequence**	**Mutants**
pp2aa1-1 _LP	AGCACATCCTTCCTTGTGTGAAGG	*pp2aa1-1*
pp2aa1-1 _RP	AACTTGCTTATGATGTTAAGGCGC	
pp2aa1-1 _RB	TGTCCCGCGTCATCGGCGGGGGTC	
*pp2aa1-6*_LP	GGCCAGCCAGTTAGGTATAGG	*pp2aa1-6*
*pp2aa 1-6*_RP	AAACATAGCCACACGCATTTC	
*pp2aa2-1*_LP	CGATGTTACGTGCCCTCTTAC	*pp2aa2-1*
*pp2aa2-1*_RP	TCTACCGAATGACCATTTTGC	
*pp2aa2-3*_LP	ATTGGTTATTTGGGATCGGAG	*pp2aa2-3*
*pp2aa2-3*_RP	ACTCTCCCTCATCTGAGAGCC	
*pp2aa3-1*_LP	TATTTCCAAACTTTGGGGGAC	*pp2aa3-1*
*pp2aa3-1*_RP	ATGGACACAGCTTGAAGATGG	
*pp2aa3-2*_LP	GCACCAAGCTTCTCATCAAAG	*pp2aa3-2*
*pp2aa3-2*_RP	GACCGGAGCCAACTAGGTAAG	
LB1	GCCTTTTCAGAAATGGATAAATAGCCTTGCTTCC	SAIL lines
LBb1.3	ATTTTGCCGATTTCGGAAC	SALK lines
pp2ac3-1-LP	GCTTGAAAGAACAGCATTTCG	*pp2ac3-1*
pp2ac3-1-RP	GTGGATTATCACCATCCATCG	
pp2ac4-1-LP	TAATTGGTATCAGGGCACTGC	*pp2ac4-1*
pp2ac4-1-RP	TGTTTCCTGATCTGTTTTCCG	
npr1-1-F	CGTGTGCTCTTCATTTCGCTGT	*npr1-1*
npr1-1-R	GTGCGGTTCTACCTTCCAAAGTT	
npr3-1-F	GAGTCAGATATCACTCTAGATCAAGC	*npr3-1*
npr3-1-R	GGAAAGAACAACTGAGCAAGCCCCA	
npr4-3-F	CGGATCTTGTTTCGTCATTTCAG	*npr4-1*
npr4-3-R	CAAACGTGAAATCTGAAGCATTAGC	
sid2-3-LP	ACCCTAATTTGGATTTGGTGC	*sid2-3*
sid2-3-RP	AGCTCTAGGCCTAGTTGCAGC	
**For Cloning**		
**Oligo Name**	**Sequence**	**Plasmid**

PIN2HL-1(EcoRI)	TGGAATTCGCTAAGCTTCTCATCTCCGAGC	pET28a-PIN2HL
PIN2HL-2(SalI)	CCGGTCGACACTCGCCGGCGGCATCTGCTG	
PP2AA1-1(EcoRI)	GGAATTCATGGCTATGGTAGATGAACCGTTG	pET28a-PP2AA1
PP2AA1-2(XhoI)	CCGCTCGAGGGATTGTGCTGCTGTGGAACCATC	
PP2AA3-1(EcoRI)	GGAATTCATGTCTATGGTTGATGAGCCTTTA	pET28a-PP2AA3
PP2AA3-2(XhoI)	CCGCTCGAGGCTAGACATCATCACATTGTC	

Table S1. List of primers used in this study. Related to STAR METHODS and Key Resources Table.
